# *EYS* is a major gene involved in retinitis pigmentosa in Japan: genetic landscapes revealed by stepwise genetic screening

**DOI:** 10.1038/s41598-020-77558-1

**Published:** 2020-11-27

**Authors:** Shogo Numa, Akio Oishi, Koichiro Higasa, Maho Oishi, Manabu Miyata, Tomoko Hasegawa, Hanako Ohashi Ikeda, Yuki Otsuka, Fumihiko Matsuda, Akitaka Tsujikawa

**Affiliations:** 1 Department of Ophthalmology and Visual Sciences, Kyoto University Graduate School of Medicine, Kyoto, Japan; 2 Department of Ophthalmology and Visual Sciences, Nagasaki University, Sakamoto 1-7-1, Nagasaki, 852-8102 Japan; 3 Department of Genome Analysis, Institute of Biomedical Science, Kansai Medical University, Osaka, Japan; 4 Kyoto Okamoto Memorial Hospital, Kyoto, Japan; 5 Center for Genomic Medicine, Kyoto University Graduate School of Medicine, Kyoto, Japan

**Keywords:** Genetic testing, Next-generation sequencing, Genetics research, Retina

## Abstract

Next-generation sequencing (NGS) has greatly advanced the studies of causative genes and variants of inherited diseases. While it is sometimes challenging to determine the pathogenicity of identified variants in NGS, the American College of Medical Genetics and Genomics established the guidelines to help the interpretation. However, as to the genetic screenings for patients with retinitis pigmentosa (RP) in Japan, none of the previous studies utilized the guidelines. Considering that *EYS* is the major causative gene of RP in Japan, we conducted stepwise genetic screening of 220 Japanese patients with RP utilizing the guidelines. Step 1–4 comprised the following, in order: Sanger sequencing for two major *EYS* founder mutations; targeted sequencing of all coding regions of *EYS;* whole genome sequencing; Sanger sequencing for *Alu* element insertion in *RP1*, a recently determined founder mutation for RP. Among the detected variants, 2, 19, 173, and 1 variant(s) were considered pathogenic and 8, 41, 44, and 5 patients were genetically solved in step 1, 2, 3, and 4, respectively. Totally, 44.5% (98/220) of the patients were genetically solved, and 50 (51.0%) were *EYS*-associated and 5 (5.1%) were *Alu* element-associated. Among the unsolved 122 patients, 22 had at least one possible pathogenic variant.

## Introduction

The global estimate of retinitis pigmentosa (RP), the most prevalent form of the inherited retinal dystrophies (IRD) across all nations and ethnicities, is 1:4000, and it is a leading cause of severe visual disabilities and blindness in developed countries^1,2^. It is clinically and genetically heterogeneous. About 100 causative genes have been identified and novel causative genes and mutations are now being reported annually^3–5^.

Recent studies have revealed the spectrum of causative genes and steadily laid the groundwork for genetic approaches to treatments for IRD^6–14^. Many clinical trials of gene therapies are also ongoing. The approval of Voretigene neparvovec as the first gene therapy for Leber congenital amaurosis (LCA)^15^, signaled the dawn of gene therapy for IRD^16–18^. Given this background, identifying causative genes and their mutations for each IRD among various ethnicities will become more important.

Next-generation sequencing (NGS) can rapidly and accurately detect variants in DNA samples from large cohorts, and this has substantially contributed to genetic screening^19–21^. However, NGS screening has led to the novel difficulty of determining the pathogenicity of enormous numbers of detected variants. The American College of Medical Genetics and Genomics (ACMG) standards and guidelines have recently become the de-facto standard criteria to address this problem^22^.

The reported genetic diagnostic rates of retinitis pigmentosa in Japan have varied, being 36.6% in 2014 by Oishi M et al^8^ and 29.6% in 2018 by Koyanagi Y et al^12^. Those previous studies were performed without considering the ACMG classifications of variants, because the ACMG guidelines had not yet been launched in 2014, and because previously reported and null variants served as the criteria for pathogenic variants in the latter study. Therefore, it would be practical to investigate whether employing the ACMG methodology would improve genetically diagnostic yield.

Moreover, considering that *EYS* is the major causative gene of RP in Japan^8,12,23–26^, we constructed the stepwise genetic screenings of patients with RP—starting with the direct Sanger sequencing for the two *EYS* founder mutations in Japan, followed by, targeted NGS for all exons of the *EYS* gene, and whole genome sequencing (WGS).

With the above research questions and the scientific backgrounds, the aim of our current study is to perform the stepwise genetic analyses for patients with RP and elucidate the landscapes of the causative genes and variants of RP in Japan using the ACMG guidelines.

## Methods

All procedures used in the current study adhered to the tenets of the Declaration of Helsinki. The Institutional Review Board of Kyoto University Graduate School of Medicine approved the study protocols (G746). All patients and their relatives were fully informed of the purpose and procedures of the study, and written consent to participate was obtained from each patient and their family member, if available.

### Study participants

The single-center study included 223 unrelated patients with RP who attended the Department of Ophthalmology at Kyoto University Hospital, Kyoto, Japan, between 2006 and 2016 and agreed to participate. An IRD specialist (O.A.) clinically diagnosed the subjects based on a history of visual symptoms and the findings of comprehensive ophthalmologic examinations. We excluded three patients with syndromic RP; two with cerebellar hypoplasia and one with mitochondrial disease (myoclonus epilepsy associated with ragged-red fibers). Finally, we analyzed data from 220 patients. Pedigrees were assembled based on detailed interviews of patients and their family members, from which inheritance patterns were inferred. With reference to a previous report^14^, if at least one family member other than the proband was affected but the pedigree was not suggestive of an autosomal-recessive (AR), autosomal-dominant (AD), X-linked (XL), or sporadic trait, we regarded the result as “inconclusive”. Genomic DNA was extracted from venous blood using QuickGene-610L DNA extraction kits (Fujifilm, Minato, Tokyo, Japan).

### Stepwise sequence analyses

**Figure 1:**
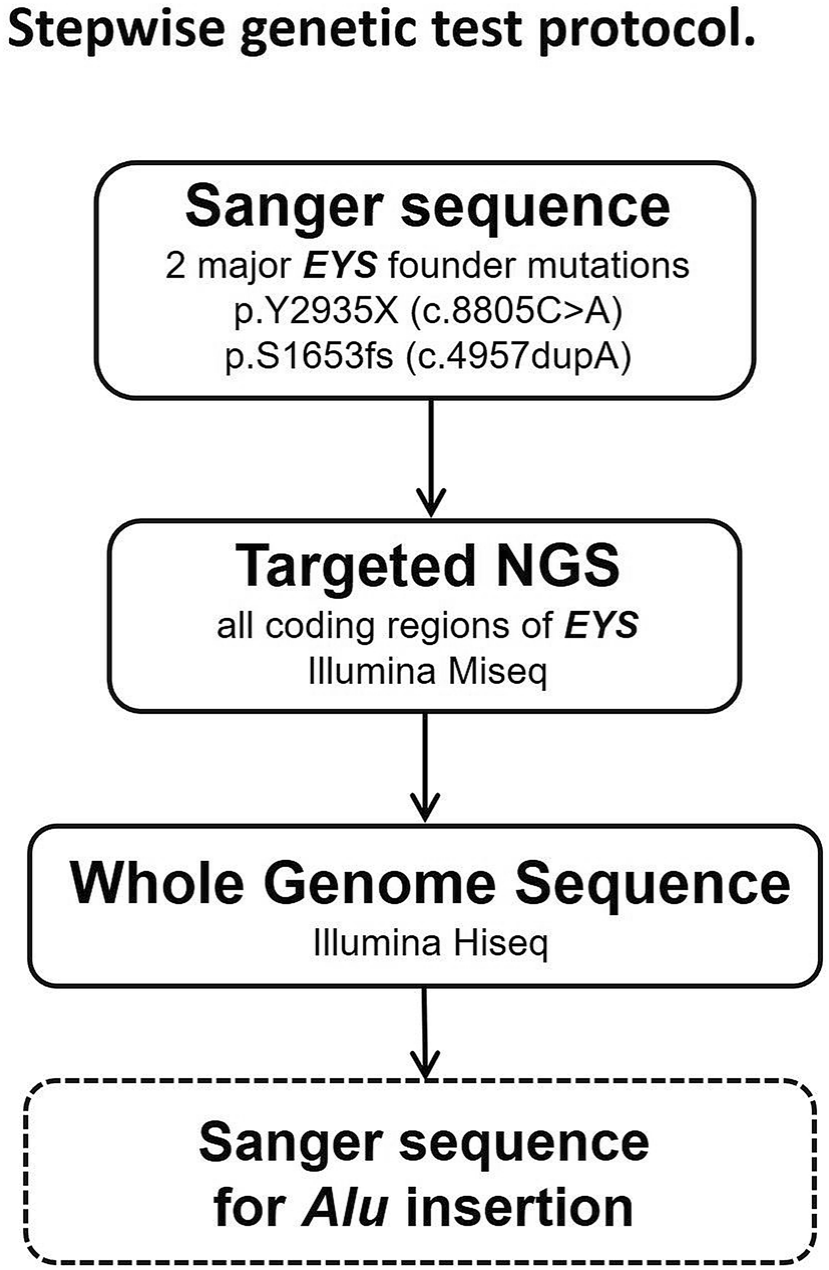
Stepwise genetic test protocol. Two frequent founder variants were initially screened in our cohort by Sanger sequencing, then *EYS* genes were screened using targeted exome sequencing. Whole genome sequencing was applied, then *Alu* insertions were screened using Sanger sequencing.

Figure 1 shows the stepwise genetic analyses, starting with Sanger sequencing for two major *EYS* founder mutations (c.8805C>A; p.Y2935X and c.4957dupA; p.S1653fs) in Japanese patients with RP. Targeted NGS for all coding regions of *EYS* was conducted by NGS using an Illumina MiSeq system (Illumina Inc., San Diego, CA, USA) for those who were not genetically solved by Sanger sequencing. We finally conducted WGS for those who were not genetically diagnosed by either of the above methods.

We then applied additional Sanger sequencing to detect the *Alu* element insertion in exon 4 of *RP1* in all analyzed patients with RP according to a recent report by Nikopoulos et al.^27^.

### Variant data analysis

A total of 10.7 kb DNA segments covering the exons of the *EYS* gene were sequenced using the Illumina MiSeq. The target regions were specifically amplified using 44 oligonucleotide pairs and the Tks Gflex enzyme (Takara Bio; Kyoto, Japan). Whole genome sequencing was conducted using an Illumina HiSeq X Ten system (Illumina Inc.). The sequence reads were aligned onto the reference genome (GRCh37/hg19) using the Burrows-Wheeler Aligner, then downstream analyses were conducted using Picard and GATK version 3.8, according to the GATK Best Practice recommendations included marking duplicates, base quality recalibration, haplotype calling, joint variant calling, and variant quality score recalibration (VQSR). Copy number variations were identified by the variance of read coverage in 5-kb bins along the reference genome sequence. Regions that were greater or less than 3/8 of the mode of the coverages were designated as alterations.

### Definition of pathogenic variants and VUS

We analyzed all variants detected in the exons and their boundaries (± 2 bps) of the genes registered for IRD in RetNet (Retinal Information Network, https://sph.uth.edu/retnet/, Supplementary Table 1), and determined pathogenic variants and variants of uncertain significance (VUS) as follows (Table 1). In the current study, we presumed only the previously-reported inheritance pattern for each gene. For example, we evaluated *SNRNP200* only for AD-trait inheritance because it is registered only for AD-trait in RetNet.

**Table 1:** Number of detected variants, pathogenic variants, and genetically solved patients in each step.

		Detected variants	Variants evaluated as pathogenic by the criteria			Genetically solved patients
			(1) "DM" or "DM?" in HGMD professional	(2) "pathogenic" or "likely pathogenic" by ACMG guidelines	(3) Large deletions or insertions	
Step1	Sanger sequence for two *EYS* founder mutations	**2**	**2**			***8***
Step2	Targeted sequencing for all *EYS* exons	**67**	**14**	**5**	**0**	***41***
Step3	Whole Genome Sequence	**1730**	**86**	**81**	**6**	***44***
Step4	Sanger sequence for *Alu* element	**1**			**1**	***5***
					**Total solved patients**	***98***

Totally 98 patients were genetically solved.

Among the detected variants in the genes registered for RP (or syndromic RP) in RetNet, we considered those satisfying at least one of the following criteria as pathogenic; 1) variants registered as “DM” or “DM?” for RP (or syndromic RP) in the Human Genome Mutation Database Professional (HGMD professional, 2019.2), but we filtered out variants with a MAF of > 1% in gnomAD even if registered in HGMD professional because RP is a rare Mendelian disease; 2) variants evaluated as “likely pathogenic” or “pathogenic” by ACMG guidelines, and 3) large deletions and insertions including an *Alu* insertion in *RP1*.

Among the detected variants in the genes registered for IRD in RetNet, we determined VUS in accordance with the criteria of ACMG guidelines.

In the current study, we considered *AIPL1*, *CRB1*, and *RCBTB1* to be causative only for the AR trait, and did not consider *FSCN2*, *GUCA1B*, *OR2W3*, *PITPNM3*, *RIMS1*, *ROM1*, *SEMA4A*, *UNC119*, and *CA4* to be causative for IRD, because their pathogenicity for the AD trait was in doubt as a result of an allele frequency analysis in a recent study by Hanany et al.^28^ (The latter group of genes; *FSCN2*, *GUCA1B*, *OR2W3*, *PITPNM3*, *RIMS1*, *ROM1*, *SEMA4A*, *UNC119*, and *CA4* were registered only in AD traits in RetNet).

Supplementary Table 1 shows the reference numbers of the genes analyzed herein.

## Results

We analyzed 220 patients (female, n = 102; male, n = 118) with RP (AD-RP, n = 4; AR-RP, n = 48; XL-RP, n = 5; sporadic RP, n = 137; inconclusive, n = 26).

We generated 5.7 Gb and 17.2 Tb DNA sequences for targeted sequencing and WGS analysis. On average, 98.7% and 99.1% of the reads were mapped to the reference sequence, which corresponded to 85.7% and 97.3%, respectively, of the target bases being covered with a depth of at least 10×.

### The first step: Sanger sequencing of two major *EYS* founder mutations

In the first step, direct Sanger sequencing for two major *EYS* founder mutations in Japan, 3, 2, and 3 patients were genetically solved with homozygous c.4957dupA (p.S1653fs), homozygous c.8805C>A (p.Y2935X), and compound heterozygous c.4957dupA (p.S1653fs) and c.8805C>A (p.Y2935X), respectively.

Totally, 8 patients were genetically solved in this step, and the 212 unsolved patients proceeded to the next step. Among the 212 patients, heterozygous c.4957dupA (p.S1653fs) and heterozygous c.8805C>A (p.Y2935X) were detected in 25 and 8 patients, respectively.

### The second step: targeted sequencing for all coding regions of *EYS*

In the second step, targeted sequencing for all coding regions of *EYS*, 67 variants were detected, and 14 and 5 variants were evaluated as pathogenic satisfying the criteria of 1) registered as causative for RP in HGMD professional 2019.2 and 2) evaluated as likely pathogenic in ACMG guidelines, respectively.

Among the 212 unsolved patients in the first step, 41 patients were genetically solved in the second step. Focusing on the 33 carriers of two founder mutations analyzed in the first step, 14 patients had compound heterozygous variants c.4957dupA (p.S1653fs) and other pathogenic variants, and 5 patients had compound heterozygous variants c.8805C>A (p.Y2935X) and other pathogenic variants.

The 171 unsolved patients proceeded to the third step of WGS.

### The third step: WGS

In the third step, WGS, 1730 variants were detected in the IRD genes shown in Supplementary Table 1, and among them, 1142 variants belonged to the genes registered as causative for RP or syndromic RP in RetNet. After filtering out by the criteria in Method section, 86, 81, and 6 out of the 1142 variants were evaluated as pathogenic satisfying the criteria of 1) registered as causative for RP in HGMD professional 2019.2, 2) evaluated as likely pathogenic in ACMG guidelines, and 3) large deletions and insertions*,* respectively. As to large deletions and insertions, read-depth analysis revealed unreported 5 large deletions and 1 large insertion in the genes registered for IRD in RetNet; 3 large deletions and 1 large insertion in *EYS*, 1 large deletion in *IQCB1*, and 1 complex rearrangement in *PRPF31*.

Among the 171 unsolved patients in the second step, 44 patients were genetically solved in the third step. Among the 6 gross changes detected by read-depth method, only two contributed to the genetic diagnoses; a heterozygous deletion that spanned exon 42 of the *EYS* and complex rearrangements including the *PRPF31*. (Supplementary Figs.1 and 2).

### The additional fourth step: *Alu* element

As the fourth step, we performed Sanger sequencing to detect the *Alu* element insertion in exon 4 of *RP1* in all 220 included patients. The results of our analysis showed that three patients each had homozygous and heterozygous *Alu* element insertions. Among the three patients with a heterozygous *Alu* element insertion, two had another pathogenic *RP1* variant and were genetically solved for AR-trait RP; one had c.4196del and the other had c.5797C>T, both of which have recently been reported^27^. Therefore, 5 patients were genetically solved in this additional step.

The allele frequency of the *Alu* element in this RP cohort was 2.0% (9 of 440 alleles), and it was ranked fifth among the most frequent variants that contributed to the genetic diagnoses for at least one patient (All the top five most frequent variants belonged to *EYS*, and the *Alu* element allele frequency was the same as the fifth most frequent *EYS* variant).

### Summaries of genetic diagnoses

Summarizing all the genetic diagnostic steps, we genetically diagnosed 98 (44.5%) of the 220 analyzed patients (Table 1 and Supplementary Table 2).

Eight patients were genetically solved just by two founder mutations in *EYS*, 41 were solved by targeted NGS of all coding regions in *EYS*, 44 were solved by WGS, and 5 were solved by additional PCR and Sanger sequencing for the *Alu* insertion in exon 4 of *RP1*. Thus, 49 (50%) of the patients were solved by the first and second steps, which searched only variants of *EYS*.

**Figure 2:**
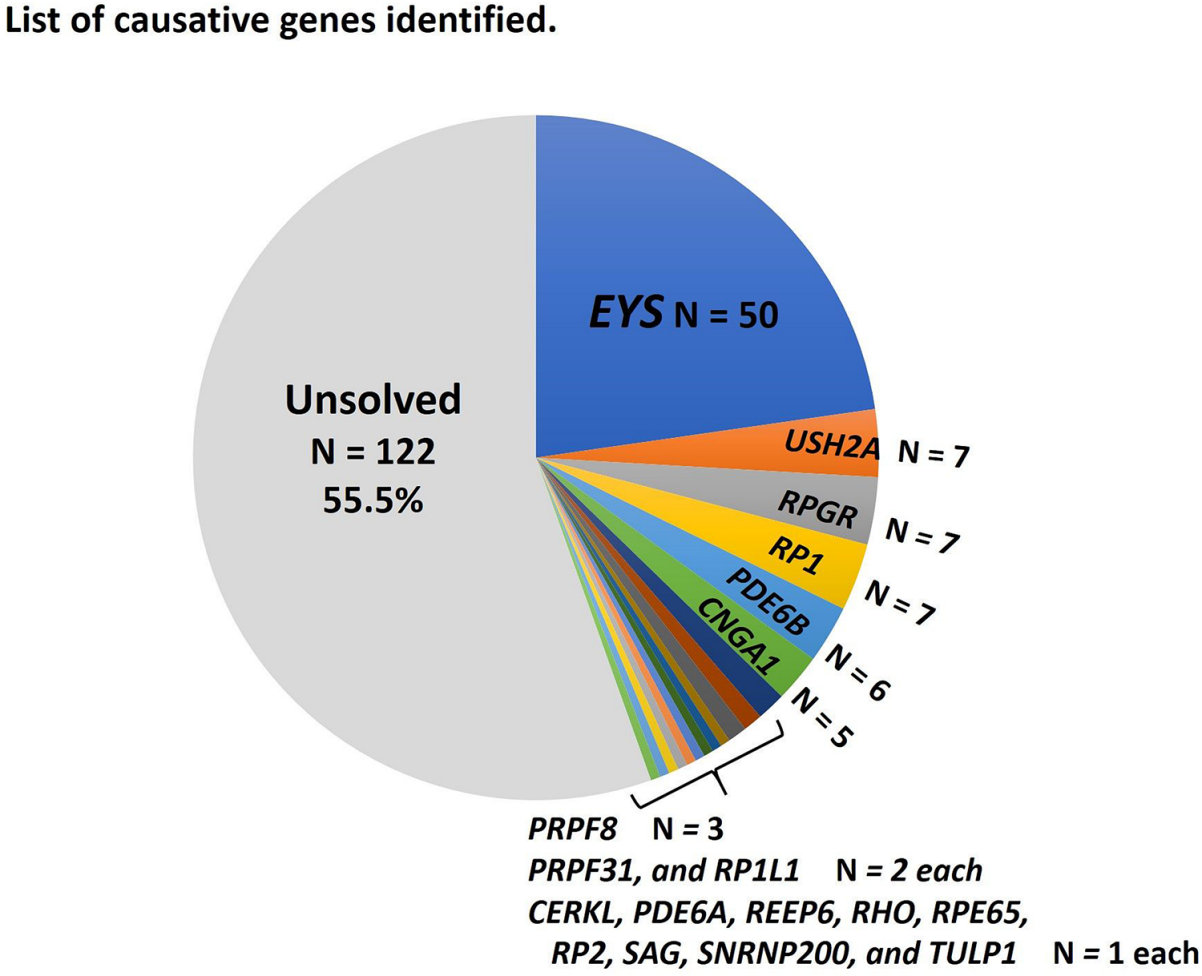
List of causative genes identified. About half of the solved patients were *EYS*-associated.

Figure 2 shows the list of causative genes in 98 genetically solved patients. The most frequent causative gene was *EYS*, and 50 (51.0%) of the genetically solved patients were *EYS*-associated. Variants in only six genes, *EYS, USH2A, RPGR, RP1, PDE6B*, and *CNGA1*, led to genetic diagnoses in 82 (83.6%) of the 98 solved patients. Supplementary Table 3 shows details of the identified causative variants.

### Details of *EYS* mutations contributing to genetic diagnoses

Adding the 67 variants detected in the second step of targeted sequencing for all coding regions of *EYS*, we detected 8 other variants in the third step of WGS; 4 variants in the exons and their boundaries, 3 large deletions and 1 large insertion. Among the total of 75 variants detected in the exons and their boundaries of *EYS* in 220 patients, 26 of them were considered pathogenic. Furthermore, 20 *EYS* variants including a single 6 kb gross deletion throughout exon 42 were causative for at least one patient (Supplementary Table 3). From the perspective of variant types, the 20 causative variants comprised five stop-gain single-nucleotide variations (SNV), six frameshift deletions, eight missense SNV, and one gross deletion. The most frequent variants in *EYS* were c.4957dupA(p.S1653fs), c.8805C>A(p.Y2935X), and c.2528G>A(p.G843E), all of which are understood to be founder mutations in Japanese populations^26^.

Fourteen causative variants of *EYS* have been registered in HGMD Professional 2019.2 as “DM” or “DM?” with published evidence, and among the other six causative variants, two (c.8268_8272del and c.8868delT) had referenced reports in which their pathogenicity was inferred. Therefore, we describe the other four variants(c.5847C>A (p.Y1949X, stopgain), c.7613delC (p.P2538fs, frameshift deletion), the 6 kb gross deletion including all of exon 42, and c.8268delC (p.S2756fs, frameshift deletion) herein as novel causative variants.

### Mild or late-onset phenotypes of patients with homozygous c.2528G>A (p.G843E) in *EYS*

Among the six patients with homozygous c.2528G>A(p.G843E) in *EYS*, four retained visual acuity ≥ 20/20 and the ratio of the patients with visual acuity ≥ 20/20 was higher than that of the 186 patients with other variants or who were genetically undiagnosed (4:2 vs. 39:147 patients with best corrected visual acuity of ≥ 20/20: < 20/20 in both eyes; P = 0.0235, Fisher exact tests). One patient who harbored homozygous c.2528G > A (p.G843E) had a wholly devastated pigmented retina and could perceive only light with both eyes at the age of 77 years. His visual acuity had been sufficient to renew his driver’s license at the age of 43 years, when a first visit to an ophthalmologist identified slight subjective symptoms. The experience of this patient indicates a late onset phenotype.

### Analysis to interpret VUS

**Figure 3:**
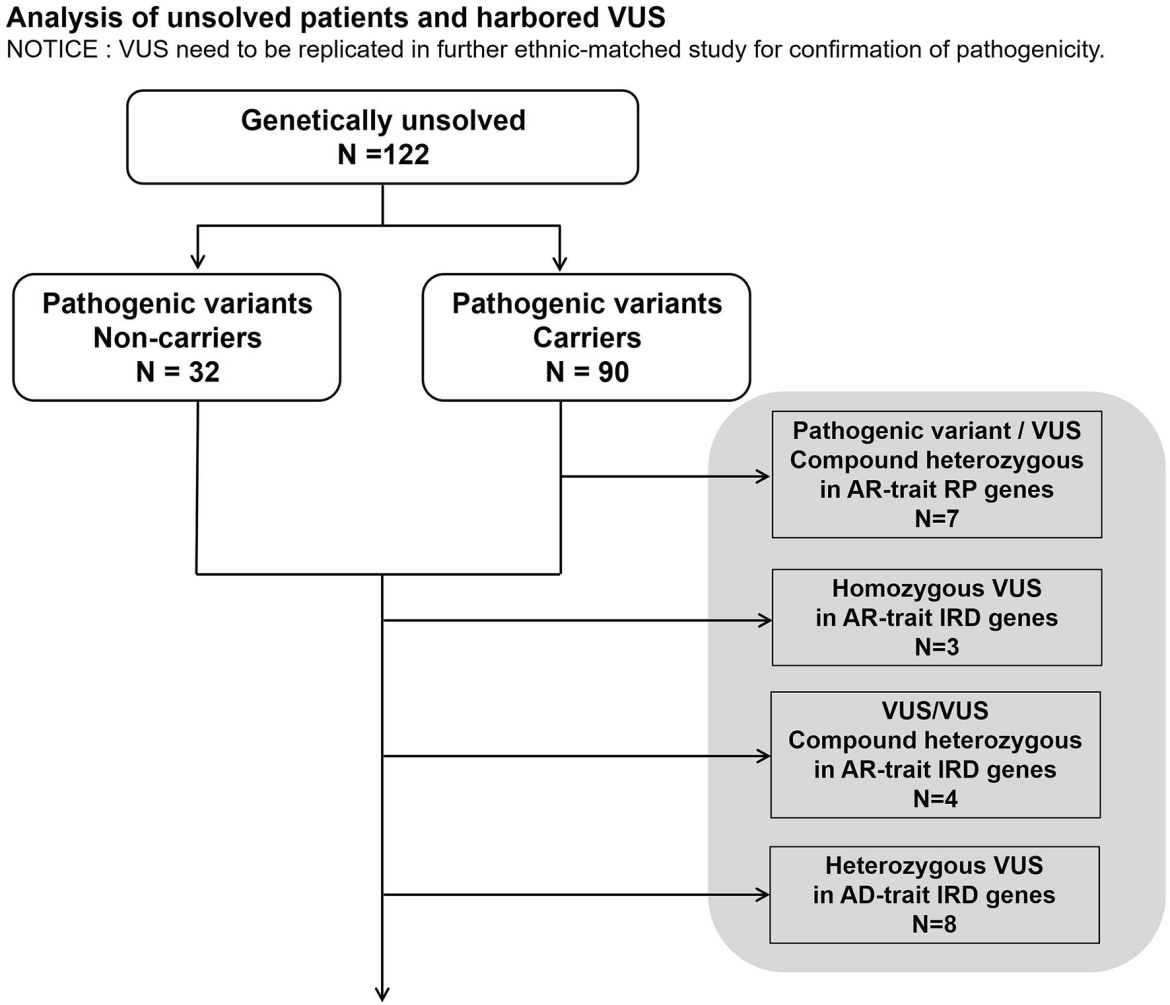
Analysis of unsolved patients and harbored VUS. Twenty-two patients had possible genetic diagnoses associated with VUS.

After the genetic diagnoses (Fig. 3), we analyzed the 122 genetically unsolved patients and their variants and found that 90 were carriers of at least one pathogenic variant in the genes registered for IRD in RetNet. Among them, seven had other variants evaluated as VUS in the same gene as the pathogenic variant. Furthermore, three of the 122 unsolved patients had homozygous VUS, eight had compound heterozygous VUS on the genes of AR-trait IRD, and four had heterozygous VUS on genes of AD-trait IRD. Overall, with respect to VUS, 22 patients were possibly genetically solved with candidate variants that need to be replicated in further study for confirmation of pathogenicity before any genetic counseling and any available clinical trials of gene therapies. The related genes were *USH2A* (n = 4), *EYS* and *SNRNP200* (n = 2 each), and *CACNA1F*, *CLN3*, *CNGA1*, *CRB1*, *FLVCR1*, *GPR98*, *GUCY2D*, *IFT140*, *MAK*, *PDE6B*, *PRPF8*, *RHO*, *TOPORS*, and *ZNF408* (n = 1 each). Among these 17 genes, 14 were registered as causative for RP or syndromic RP in RetNet, and *CLN3*, *GUCY2D*, and *CACNA1F*, were registered as causative for other IRD. Supplementary Tables 4 and 5 show details of the patients and the involved VUS.

### Atypical phenotypes of possibly solved patients with candidate variants in RP genes that need to be replicated in further study for confirmation of pathogenicity

Among the 19 possibly solved patients with VUS, candidate variants that need to be replicated in further study, in the genes registered as causative for RP or syndromic RP, three had atypical phenotypes. Supplementary Fig. 3 shows the phenotype of a female patient with a homozygous VUS of *CNGA1* (c.41A>T). Dystrophy was limited to the nasal area, and her macula region and temporal retina were almost normal in both eyes. Full-field electroretinography (ffERG) showed essentially undetectable rod and subnormal cone responses despite having temporal retina in almost normal state. Supplementary Fig. 4 shows the phenotype of a male patient with a compound heterozygous VUS of *GPR98* (c.940G>A and c.3046G>A), who lacked characteristic peripheral bone-spicule-like pigmentary changes (retinitis pigmentosa sine pigmento). Supplementary Fig. 5 shows the atypical phenotype of a female patient with a heterozygous VUS of *CRB1* (c.3131C>T) for the AD trait. She had bilaterally symmetrical paravenous dystrophy, which is obvious in autofluorescence images of the fundus, and essentially undetectable rod and cone responses in ffERG.

### Phenotypes of possibly solved patients with candidate variants in non-RP IRD genes that need to be replicated in further study for confirmation of pathogenicity

Supplementary Fig. 6 shows the phenotype of a male patient with a homozygous VUS of *CLN3* (c.1007C > A). He had a dystrophic retina without pigmentation (retinitis pigmentosa sine pigmento), and his rod and cone responses in ffERG were non-recordable at the age of 26 years. Supplementary Fig. 7 shows the phenotype of a male patient with a heterozygous VUS of *GUCY2D* (c.162C>G). He had a typically dystrophic retina with pigment near the arcade vessel, and his rod and cone responses were subnormal at the age of 64 years. A female patient with a compound heterozygous VUS of *CACNA1F* (c.3334G>T and c.3346 T>G) had the typical phenotype of diffuse pigment and narrowed vessels in the fundus, non-recordable rod and essentially undetectable cone responses in ffERG. However, her medical records contained only poor-quality images.

## Discussion

We conducted stepwise genetic screening for Japanese patients with RP using direct Sanger sequencing for the two *EYS* founder mutations in Japan, targeted NGS of all coding regions of *EYS*, WGS, and additional Sanger sequencing for the *Alu* element insertion in *RP1*, and determined genetic diagnoses in 98 (44.5%) of 220 analyzed patients. The six major causative genes, *EYS, USH2A, RPGR, RP1, PDE6B,* and *CNGA1*, were responsible for 83.6% of the diagnosed patients.

Since the first description of the pathogenic effect of *EYS* variants on RP in 2008^29,30^, many studies have revealed the prevalence of *EYS*-associated RP in patients with various ethnicities: 15.9% AR traits among those in Spain^31^, 12% sporadic or AR traits in those from France^32^, 11% in UK and China^33^, 10% in Germany , 9.1% in Korea^34^, 7% in Israel^35^, 5% in The Netherlands^36^, and 0% in Northern Ireland^9^. The present findings reconfirmed the significant contribution of *EYS* to genetic diagnoses for Japanese patients with RP. Among our 98 genetically solved patients, *EYS* accounted for 50 (51.0%) of them, and only five *EYS* variants (c.2528G>A, c.4957dupA, c.6557G>A, c.7919G>A, and c.8805C>A) accounted for 38 (38.8%). The results of our analysis showed that including the three *EYS* mutations; c.2528G>A, c.6557G>A, and c.7919G>A, into the two well-known *EYS* founder mutations would improve the rate of genetic diagnoses by Sanger sequence in Japanese patients with RP.

The most frequent *EYS* variants in our cohort were c.2528G>A (*EYS* v1), c.4957dupA (*EYS* v2), and c.8805C>A (*EYS* v3) with allele frequencies of 10.4%, 7.7%, and 3.4%, respectively. The founder mutations, *EYS* v2 and *EYS* v3, are frequently reported in Japan^8,23–26^. Even though the pathogenicity of *EYS* v1 has remained controversial due to its high prevalence in Japanese public databases (2.2% in HGVD and 1.7% in ToMMo 4.7KJPN), we considered it pathogenic, as reported by Iwanami et al.^26^. They found the missense variant segregated with RP in 10 families and included five patients who harbored the homozygous variant. In agreement with these findings, the allele frequency of the variant in patients with RP was significantly and statistically higher than that in controls (P < 0.0001 chi-squared tests). Iwanami et al. also referred to the possibility of a mild or late-onset phenotype in patients with *EYS* v1, and our findings mentioned in Results section supported that notion.

We detected the *Alu* element insertion including a premature termination codon in the coding region (p.Y1352A fs*9) in six patients and genetically solved five (2.3% of the analyzed patients) as having the *RP1 Alu-associated RP* for the AR trait. Considering its high allelic frequency in Japanese patients with RP (2.0%) and its significant contribution to genetic screening, it might be a reasonable strategy to screen this large insertion in addition to the five frequent *EYS* variants determined by direct Sanger sequencing before whole exome sequencing (WES) or WGS. Nikopoulos et al. reported that the *Alu* element insertion, *RP1* frameshift variant c.4196del (p.C1399Lfs*5) and the *RP1* stop-gain variant c.5797C>T (p.R1933*) were not pathogenic for AD-RP when heterozygous. Furthermore, the *RP1* stop-gain variant c.5797C>T (p.R1933*) was benign in the homozygous form but pathogenic with *Alu* element insertion in the compound heterozygous form^27^. This non-Mendelian pattern of inheritance might explain the genetically undiagnosed patients to some extent as well as novel causative genes, deep-intronic variants^37–39^, and medium-to-large deletions and insertions as well as structural variants, that are difficult to detect by WES or WGS using short-read sequencers^40–42^.

Figure 3 shows that 22 out of the 122 genetically unsolved patients were possibly genetically solved with the candidate causative variants. We must add prudent and cautious comments on these candidate variants shown in Supplementary Table 5. These variants satisfied the criteria of ACMG guidelines not for pathogenic variants but for VUS, therefore they need to be replicated in further study for confirmation of pathogenicity before the patients of RP with the candidate variants are recruited to any available clinical trials of gene therapies.

Of note, the atypical phenotypes of the patients who were possibly genetically solved with the candidate variants that need to be replicated in further study resembled those that were previously reported. For example, the phenotype of our patient with a homozygous VUS of *CLN3* (Supplementary Fig. 6), was similar to that of five patients with unpigmented atypical RP described by Ku C A et al. (2017)^43^. Another example is the atypical paravenous pigmented phenotype of our patient having heterozygous *CRB1* (Supplementary Fig. 5). *CRB1* is known to be causative for pigmented paravenous retinochoroidal atrophy, which is similar to our case. Of course, the pathogenicity of these VUS is very difficult to verify because most of the variants have not been functionally studied, animal models have yet to supply confirmatory data and in silico prediction programs are just supportive but not decisive. More controls, patients with RP, and accumulated illustrative familial data are essential to determine the pathogenicity of the VUS listed in Supplementary Table 5 using the evidence framework of the ACMG guidelines.

The rate of genetic diagnosis herein was 44.5%, which was an increase of 7.9% compared with the previous reports from our institute in 2014^8^. The following factors might explain the difference. Although previously regarded as non-pathogenic, we considered the most frequently causative variant, *EYS* v1, as pathogenic based on more evidence^25,26^, the criteria of the ACMG guidelines^22^ that were launched in 2015, and new causative variants of RP, including *Alu* element insertion in *RP1* that have recently been identified^27^.

This study has several limitations that might have affected the rate of the genetic diagnoses. Short-read sequencing could not detect some types of variants, such as gross structural variants and variants in long repeating regions. Novel third generation, long-read sequencing would contribute to the genetic diagnoses of patients who were hitherto genetically undiagnosed, especially carriers of pathogenic AR-RP variants^40–42^. We assessed previous findings considering the deleterious effects of the detected variants, but we did not fully analyze conflicting reports suggesting that the variants might be benign. However, the various methods applied in previous studies were not standardized. Therefore, we did not include evidence for benignancy in the current study. We did not conduct segregation analyses due to the lack of familial samples and data. Considering that segregation data are highly valued in ACMG guidelines and are regarded as a “Strong” level evidence when increased, the accumulation of not only samples from patients but also their families will be very important for further determinations of variant pathogenicity.

In conclusion, we identified causative variants in 98 (44.5%) of 220 Japanese patients with RP by stepwise genetic screening. Over half of the solved patients were *EYS*-associated, and our stepwise screening protocol functioned well for this cohort. We detected *Alu* element insertions in the *RP1* genes of six patients, and the allelic frequency was 2.0%. Applying the ACMG guidelines to evaluate unreported variants and the steady accumulation of IRD and familial samples will contribute to more accurate genetic diagnoses and be of considerable importance to patients with inherited visual defects in the era of gene therapy.

## Supplementary information

Supplementary information.

## Data Availability

All data generated/analyzed in this study are included in this article or in the Supplementary Information files and can be provided upon request.
