# Supplementary material for: EYS is a major gene involved in retinitis pigmentosa in Japan: genetic landscapes revealed by stepwise genetic screening
Source: Sci Rep. 2020 Nov 27;10:20770. doi: 10.1038/s41598-020-77558-1 (PMC7695703; doi:10.1038/s41598-020-77558-1)
Supplement: Supplementary file 1 — Supplementary information. [file 41598_2020_77558_MOESM1_ESM.pdf]

EYS is a major gene involved in retinitis pigmentosa in Japan: Genetic landscapes revealed by stepwise genetic screening

Shogo Numa[1], Akio Oishi[2], Koichiro Higasa[3], Maho Oishi[1,4], Manabu Miyata[1], Tomoko Hasegawa[1], Hanako Ohashi Ikeda[1], Yuki Otsuka[1], Fumihiko Matsuda[5], Akitaka Tsujikawa[1]

1 Department of Ophthalmology and Visual Sciences, Kyoto University Graduate School of Medicine, Kyoto, Japan
2 Department of Ophthalmology and Visual Sciences, Nagasaki University, Nagasaki, Japan
3 Department of Genome Analysis, Institute of Biomedical Science, Kansai Medical University, Osaka, Japan
4 Kyoto Okamoto Memorial Hospital, Kyoto, Japan
5 Center for Genomic Medicine, Kyoto University Graduate School of Medicine, Kyoto, Japan

Supplementary Table 1. Reference numbers of the analyzed genes.
Supplementary Table 2. Details of genetically solved patients and causative variants.
Supplementary Table 3. Details of causative variants.
Supplementary Table 4. Details of possibly solved patients, causative variants and VUS.
Supplementary Table 5. Details of candidate causative variants evaluated as VUS in the current study.
Supplementary Figure 1. Heterozygous deletion spanning exon 42 of *EYS*.
Supplementary Figure 2. Complex rearrangements including *PRPF31*.
Supplementary Figure 3. Atypical phenotype of patient possibly solved with VUS of *CNGA1*.
Supplementary Figure 4. Atypical phenotype of patient possibly solved with VUS of *GPR98.*
Supplementary Figure 5. Atypical phenotype of patient possibly solved with VUS of *CRB1.*
Supplementary Figure 6. Atypical phenotype of patient possibly solved with VUS of *CLN3.*
Supplementary Figure 7. Phenotype of the patient possibly solved with VUS of *GUCY2D.*

NOTICE :
The variants listed in Supplementary Table 4 and 5 need to be replicated in further ethnic-matched study for confirmation of pathogenicity before the patients are recruited to any available clinical trials of gene therapies.

Supplementary Table 1. Reference numbers of the analyzed genes.

Gene	NM number	Gene	NM number	Gene	NM number	Gene	NM number
ABCA4	NM_000350	*C1QTNF5*	NM_015645	*CNGA3*	NM_001298	*GUCY2D*	NM_000180
ABHD12	NM_001042472	*C21orf2*	NM_004928	*CNGB1*	NM_001297	*HARS*	NM_002109
ADAM9	NM_003816	*C2orf71*	NM_001029883	*CNGB3*	NM_019098	*HGSNAT*	NM_152419
ADAMTS18	NM_199355	*C8orf37*	NM_177965	*CNNM4*	NM_020184	*HK1*	NM_000188
AGBL5	NM_021831	*CABP4*	NM_145200	*CRB1*	NM_201253	*HMCN1*	NM_031935
AHI1	NM_017651	*CACNA1F*	NM_005183	*CRX*	NM_000554	*HMX1*	NM_018942
AHR	NM_001621	*CACNA2D4*	NM_172364	*CSPP1*	NM_024790	*IDH3B*	NM_006899
AIPL1	NM_014336	*CAPN5*	NM_004055	*CTNNA1*	NM_001903	*IFT140*	NM_014714
ALMS1	NM_015120	*CC2D2A*	NM_001080522	*CYP4V2*	NM_207352	*IFT172*	NM_015662
ARHGEF18	NM_001130955	*CCT2*	NM_006431	*DFNB31*	NM_015404	*IFT27*	NM_006860
ARL2BP	NM_012106	*CDH23*	NM_022124	*DHDDS*	NM_024887	*IFT81*	NM_014055
ARL3	NM_004311	*CDH3*	NM_001793	*DHX38*	NM_014003	*IMPDH1*	NM_000883
ARL6	NM_177976	*CDHR1*	NM_033100	*DRAM2*	NM_178454	*IMPG1*	NM_001563
ARSG	NM_014960	*CEP19*	NM_032898	*ELOVL4*	NM_022726	*IMPG2*	NM_016247
ATXN7	NM_000333	*CEP250*	NM_007186	*ERCC6*	NM_000124	*INPP5E*	NM_019892
BBIP1	NM_001195306	*CEP290*	NM_025114	*ESPN*	NM_031475	*INVS*	NM_014425
BBS1	NM_024649	*CEP78*	NM_001098802	*EXOSC2*	NM_014285	*IQCB1*	NM_001023570
BBS10	NM_024685	*CERKL*	NM_001030311	*EYS*	NM_001142800	*ITM2B*	NM_021999
BBS12	NM_152618	*CHM*	NM_000390	*FAM161A*	NM_001201543	*JAG1*	NM_000214
BBS2	NM_031885	*CIB2*	NM_006383	*FLVCR1*	NM_014053	*KCNJ13*	NM_002242
BBS4	NM_033028	*CLCC1*	NM_001048210	*GDF6*	NM_001001557	*KCNV2*	NM_133497
BBS5	NM_152384	*CLN3*	NM_001042432	*GNPTG*	NM_032520	*KIAA1549*	NM_001164665
BBS7	NM_176824	*CLRN1*	NM_174878	*GPR98*	NM_032119	*KLHL7*	NM_001031710
BBS9	NM_198428	*CLUAP1*	NM_015041	*GPR125*	NM_145290	*LAMA1*	NM_005559
BEST1	NM_004183	*CNGA1*	NM_000087	*GUCA1A*	NM_000409	*LCA5*	NM_181714

Supplementary Table 1. Reference numbers of the analyzed genes.

Gene	NM number	Gene	NM number	Gene	NM number	Gene	NM number
LRAT	NM_004744	*PANK2*	NM_153638	*PRPH2*	NM_000322	*SDCCAG8*	NM_006642
LZTFL1	NM_020347	*PCDH15*	NM_033056	*RAB28*	NM_004249	*SLC7A14*	NM_020949
MAK	NM_001242957	*PCYT1A*	NM_005017	*RAX2*	NM_032753	*SNRNP200*	NM_014014
MAPKAPK3	NM_001243926	*PDE6A*	NM_000440	*RBP3*	NM_002900	*SPARC*	NM_003118
MERTK	NM_006343	*PDE6B*	NM_000283	*RCBTB1*	NM_018191	*SPATA7*	NM_018418
MFSD8	NM_152778	*PDE6C*	NM_006204	*RD3*	NM_183059	*SPP2*	NM_006944
MKKS	NM_018848	*PEX1*	NM_000466	*RDH11*	NM_016026	*TMEM216*	NM_001173990
MKS1	NM_017777	*PEX2*	NM_000318	*RDH12*	NM_152443	*TMEM237*	NM_001044385
MVK	NM_000431	*PEX7*	NM_000288	*RDH5*	NM_002905	*TOPORS*	NM_005802
MYO7A	NM_000260	*PGK1*	NM_000291	*REEP6*	NM_001329556	*TRIM32*	NM_012210
NBAS	NM_015909	*PHYH*	NM_006214	*RGR*	NM_001012720	*TRNT1*	NM_182916
NEK2	NM_002497	*PNPLA6*	NM_006702	*RHO*	NM_000539	*TTC8*	NM_198309
NEUROD1	NM_002500	*POC1B*	NM_172240	*RLBP1*	NM_000326	*TTLL5*	NM_015072
NMNAT1	NM_022787	*POC5*	NM_001099271	*RP1*	NM_006269	*TTPA*	NM_000370
NPHP1	NM_000272	*POMGNT1*	NM_017739	*RP1L1*	NM_178857	*TUB*	NM_003320
NPHP3	NM_153240	*PRCD*	NM_001077620	*RP2*	NM_006915	*TULP1*	NM_003322
NPHP4	NM_015102	*PRDM13*	NM_021620	*RP9*	NM_203288	*USH1C*	NM_005709
NR2E3	NM_014249	*PROM1*	NM_006017	*RPE65*	NM_000329	*USH1G*	NM_173477
OAT	NM_000274	*PRPF3*	NM_004698	*RPGR*	NM_000328	*USH2A*	NM_206933
OFD1	NM_003611	*PRPF31*	NM_015629	*RPGRIP1*	NM_020366	*WDPCP*	NM_015910
OPN1LW	NM_020061	*PRPF4*	NM_004697	*RPGRIP1L*	NM_015272	*ZNF408*	NM_024741
OPN1SW	NM_001708	*PRPF6*	NM_012469	*SAG*	NM_000541	*ZNF423*	NM_015069
OTX2	NM_172337	*PRPF8*	NM_006445	*SAMD11*	NM_152486	*ZNF513*	NM_144631

Supplementary Table 2. Details of genetically solved patients and causative variants.

SampleID	Sex	Age of onset	First subjective symptom	Age of first visit to our institute	VA at first visit to our institute	Hereditary trait indicated from pedigree tree	Gene	Genotype	Variant 1	Variant 2	Variant 3	Comment on phenotype
RPKT0332	M	46	constriction of VF	46	1.2/1.0	inconclusive	*EYS*	compound hetero	c.2528G>A:p.G843E	c.8860T>C:p.F2954L	-	combinded with NTG
RPKT0334	M	childhood	night blindness	41	0.7/0.8	inconclusive	*EYS*	homo	c.2528G>A:p.G843E	c.2528G>A:p.G843E	-	combined with granular corneal dystrophy
RPKT0341	F	childhood	p/o at occasional medical exam	35	0.4/0.3	sporadic	*EYS*	compound hetero	c.3809T>G:p.V1270G	c.4957dupA:p.S1653fs	c.8268delC:p.S2756fs	n.p.
RPKT0351	F	43	night blindness	57	0.6/0.6	AR	*EYS*	compound hetero	c.2528G>A:p.G843E	c.4957dupA:p.S1653fs	-	n.p.
RPKT0358	M	childhood	decreased VA	26	0.6/0.9	sporadic	*PDE6B*	compound hetero	c.1768delA:p.M590fs	c.1920+2T>C:p.?	-	n.p.
RPKT0359	F	29	decreased VA	51	0.4/1.0	AR	*EYS*	homo	c.6557G>A:p.G2186E	c.6557G>A:p.G2186E	-	n.p.
RPKT0360	M	**not recorded**	night blindness	59	0.04/0.8	AR	*CNGA1*	homo	c.265delC:p.L89fs	c.265delC:p.L89fs	-	n.p.
RPKT0362	F	41	night blindness	46	1.2/1.2	AR	*EYS*	compound hetero	c.4957dupA:p.S1653fs	c.8805C>A:p.Y2935X	-	n.p.
RPKT0364	M	**not recorded**	**not recorded**	58	0.1/0.7	AR	*EYS*	homo	c.4957dupA:p.S1653fs	c.4957dupA:p.S1653fs	-	n.p.
RPKT0370	M	44	p/o at occasional medical exam	59	0.8/0.6	AR	*EYS*	compound hetero	c.1750G>T:p.E584X	c.2528G>A:p.G843E	-	n.p.
RPKT0372	F	childhood	night blindness	26	0.6/0.7	AR	*EYS*	homo	c.4957dupA:p.S1653fs	c.4957dupA:p.S1653fs	-	n.p.
RPKT0377	M	30	night blindness	58	0.6/0.7	sporadic	*EYS*	homo	c.7919G>A:p.W2640X	c.7919G>A:p.W2640X	-	n.p.
RPKT0380	F	in her 5th decades	constriction of VF	53	1.5/1.2	sporadic	*RP1L1*	homo	c.3026_3029del:p.1009_1010del	c.3026_3029del:p.1009_1010del	-	n.p.
RPKT0382	M	adolescence	night blindness	63	0.7/0.6	inconclusive	*PDE6B*	homo	c.1669C>T:p.H557Y	c.1669C>T:p.H557Y	-	n.p.
RPKT0399	M	childhood	night blindness	63	0.06/0.05	sporadic	*RPE65*	homo	c.1543C>T:p.R515W	c.1543C>T:p.R515W	-	n.p.
RPKT0401	F	34	night blindness	54	0.2/0.2	AR	*USH2A*	compound hetero	c.490G>T:p.V164F	c.13010C>T:p.T4337M	-	n.p.
RPKT0404	F	31	color vision deficienvy	31	1.5/1.5	AR	*USH2A*	homo	c.8254G>A:p.G2752R	c.8254G>A:p.G2752R	-	n.p.
RPKT0406	F	53	p/o at occasional medical exam	63	0.7/1.5	sporadic	*RHO*	hetero	c.302G>A:p.G101E	-	-	n.p.
RPKT0409	F	40	night blindness	58	0.1/0.1	AR	*EYS*	homo	c.7919G>A:p.W2640X	c.7919G>A:p.W2640X	-	n.p.
RPKT0411	M	childhood	night blindness	8	1.5/1.5	sporadic	*PDE6B*	compound hetero	c.1604T>A:p.I535N	c.1669C>T:p.H557Y	-	n.p.
RPKT0413	F	40	p/o at occasional medical exam	41	1.5/1.5	sporadic	*EYS*	homo	c.2528G>A:p.G843E	c.2528G>A:p.G843E	-	n.p.
RPKT0418	M	44	night blindness	51	0.2/0.1	sporadic	*EYS*	compound hetero	c.2528G>A:p.G843E	c.8805C>A:p.Y2935X	-	n.p.
RPKT0423	F	childhood	night blindness	63	SL+/HM	sporadic	*EYS*	homo	c.4957dupA:p.S1653fs	c.4957dupA:p.S1653fs	-	n.p.
RPKT0437	M	15	night blindness	22	1.0/1.0	sporadic	*EYS*	compound hetero	c.2528G>A:p.G843E	c.6557G>A:p.G2186E	-	n.p.
RPKT0439	F	childhood	night blindness	60	0.04/HM	AR	*PDE6B*	homo	c.1604T>A:p.I535N	c.1604T>A:p.I535N	-	n.p.
RPKT0440	F	childhood	night blindness	29	1.0/0.9	sporadic	*PDE6A*	homo	c.166G>T:p.E56X	c.166G>T:p.E56X	-	n.p.
RPKT0442	M	30	night blindness	42	1.5/1.5	sporadic	*PRPF31*	hetero	c.316G>T:p.E106X	-	-	n.p.
RPKT0444	M	childhood	night blindness	33	0.7/1.0	sporadic	*EYS*	compound hetero	c.6557G>A:p.G2186E	c.8650T>C:p.C2884R	-	n.p.
RPKT0453	M	70	decreased VA	89	0.6/0.5	sporadic	*EYS*	compound hetero	c.2528G>A:p.G843E	c.8805C>A:p.Y2935X	-	n.p.
RPKT0456	M	childhood	night blindness	53	0.02/0.2	inconclusive	*RPGR*	hemi	c.793dupA:p.T265fs	-	-	n.p.
RPKT0461	M	6	decreased VA	26	0.6/0.6	sporadic	*RPGR*	hemi	c.2236_2237del:p.746_746del	-	-	n.p.
RPKT0462	M	in his 4th decades	night blindness	45	1.0/1.2	sporadic	*USH2A*	compound hetero	c.2802T>G:p.C934W	c.9799T>C:p.C3267R	-	n.p.
RPKT0467	F	20	decreased VA	52	SL+/SL+	AR	*RP1*	homo*	c.4196delG:p.C1399fs	c.6353G>A:p.S2118N	-	n.p.
RPKT0472	M	childhood	night blindness	21	0.7/0.9	XL	*RPGR*	hemi	c.1665delA:p.E555fs	-	-	n.p.
RPKT0473	F	42	night blindness	44	0.8/0.9	sporadic	*USH2A*	compound hetero	c.2802T>G:p.C934W	c.7660C>T:p.Q2554X	-	n.p.
RPKT0474	M	childhood	night blindness	18	0.4/0.6	AR	*RP1*	homo	c.4052_4053ins328	c.4052_4053ins328	-	n.p.
RPKT0477	M	38	night blindness	38	1.0/1.2	inconclusive	*RPGR*	hemi	c.764C>T:p.T255I	-	-	n.p.
RPKT0478	M	childhood	night blindness	24	0.04/0.04	sporadic	*RP1*	compound hetero	c.4052_4053ins328	c.4196delG:p.C1399fs	c.6353G>A:p.S2118N	n.p.
RPKT0479	F	20	night blindness	47	0.5/0.6	AR	*EYS*	homo	c.2528G>A:p.G843E	c.2528G>A:p.G843E	-	n.p.
RPKT0481	F	58	p/o at occasional medical exam	58	0.9/1.0	sporadic	*EYS*	homo	c.6557G>A:p.G2186E	c.6557G>A:p.G2186E	-	combined with PACS
RPKT0482	M	44	decreased VA	44	0.03/0.01	AD	*PRPF8*	hetero	c.5792C>T:p.T1931M	-	-	n.p.
RPKT0485	F	in her 5th decades	night blindness	63	0.02/0.06	AR	*USH2A*	homo	c.10859T>C:p.I3620T	c.10859T>C:p.I3620T	-	n.p.
RPKT0487	F	childhood	night blindness	29	0.3/0.5	sporadic	*RP1*	compound hetero	c.4052_4053ins328	c.5797C>T:p.R1933X	-	n.p.
RPKT0490	F	39	night blindness	41	0.4/1.0	sporadic	*EYS*	compound hetero	c.2528G>A:p.G843E	c.4957dupA:p.S1653fs	-	n.p.
RPKT0492	M	41	p/o at occasional medical exam	59	HM/0.02	sporadic	*EYS*	compound hetero	c.2528G>A:p.G843E	c.4957dupA:p.S1653fs	-	combined with NTG
RPKT0495	M	childhood	night blindness	54	0.8/0.6	AR	*CNGA1*	compound hetero	c.191delG:p.G64fs	c.265delC:p.L89fs	-	n.p.
RPKT0496	F	childhood	night blindness	61	0.02/HM	AR	*CNGA1*	homo	c.265delC:p.L89fs	c.265delC:p.L89fs	-	n.p.
RPKT0499	F	childhood	night blindness	10	1.2/1.2	sporadic	*PDE6B*	compound hetero	c.1486delC:p.P496fs	c.1604T>A:p.I535N	-	n.p.

NTG: normal tension glaucoma, PACS: primary angle closure syndrome, n.p.: nothing particular, p/o: pointed out, RD: retinal detachment, VF: visual field
*RPKT0467 was homozygous for two variants c.4196delG and c.6353G>A. **Gross deletion shown in Supplementary Figure1
***Complex rearrangement comprising gross deletion shown in Supplementary Figure2

Supplementary Table 2. Details of genetically solved patients and causative variants.

RPKT0501	F	30	photophibia	31	1.2/0.9	sporadic	*EYS*	compound hetero	c.2528G>A:p.G843E	c.8805C>A:p.Y2935X	-	n.p.
RPKT0507	F	46	photophibia	70	0.9/0.7	sporadic	*EYS*	compound hetero	c.3489T>A:p.N1163K	c.3809T>G:p.V1270G	-	combined with NTG
RPKT0514	M	10	night blindness	11	1.2/1.2	sporadic	*PRPF8*	hetero	c.6926A>C:p.H2309P	-	-	n.p.
RPKT0516	M	adolescence	night blindness	66	Sl+/SL+	sporadic	*EYS*	homo	c.2528G>A:p.G843E	c.2528G>A:p.G843E	-	n.p.
RPKT0517	M	in her 5th decades	decreased VA	66	0.5/0.3	AR	*USH2A*	homo	c.10859T>C:p.I3620T	c.10859T>C:p.I3620T	-	n.p.
RPKT0521	F	adolescence	night blindness	24	1.0/0.6	sporadic	*RP1*	homo	c.4052_4053ins328	c.4052_4053ins328	-	n.p.
RPKT0522	F	childhood	night blindness	58	0.8/0.7	sporadic	*CNGA1*	compound hetero	c.191delG:p.G64fs	c.265delC:p.L89fs	-	n.p.
RPKT0523	M	54	night blindness	60	0.2/0.1	AR	*PDE6B*	homo	c.51delT:p.D17fs	c.51delT:p.D17fs	-	n.p.
RPKT0525	F	58	night blindness	60	1.0/1.2	sporadic	*EYS*	compound hetero	c.2528G>A:p.G843E	c.2886C>G:p.F962L	c.2892A>C:p.E964D	n.p.
RPKT0527	M	childhood	night blindness	10	0.9/0.9	AR	*RPGR*	hemi	c.2236_2237del:p.746_746del	-	-	n.p.
RPKT0528	M	35	p/o at occasional medical exam	35	1.5/1.5	sporadic	*EYS*	compound hetero	c.4957dupA:p.S1653fs	gross deletion including exon42**	-	n.p.
RPKT0534	M	20	night blindness	59	0.5/0.4	AR	*EYS*	compound hetero	c.4897A>G:p.K1633E	c.7609G>A:p.A2537T	-	n.p.
RPKT0535	F	adolescence	night blindness	29	1.2/1.5	sporadic	*EYS*	compound hetero	c.2528G>A:p.G843E	c.4957dupA:p.S1653fs	-	n.p.
RPKT0537	M	adolescence	night blindness	39	0.7/0.7	sporadic	*RP1L1*	homo	c.324_325insT:p.P109fs	c.324_325insT:p.P109fs	-	n.p.
RPKT0539	M	52	decreased VA	52	0.6/1.2	sporadic	*EYS*	compound hetero	c.2528G>A:p.G843E	c.7919G>A:p.W2640X	-	n.p.
RPKT0541	M	27	p/o at occasional medical exam	39	1.5/1.5	sporadic	*EYS*	compound hetero	c.2528G>A:p.G843E	c.4957dupA:p.S1653fs	-	n.p.
RPKT0544	M	in his 3rd decades	decreased VA	31	0.9/0.8	sporadic	*EYS*	compound hetero	c.2528G>A:p.G843E	c.7665_7666del:p.2555_2556del	-	combined with left eye interstitial keratitis
RPKT0549	M	childhood	night blindness	66	0.9/0.7	sporadic	*CNGA1*	homo	c.191delG:p.G64fs	c.191delG:p.G64fs	-	n.p.
RPKT0552	F	childhood	night blindness	9	1.5/1.5	sporadic	*EYS*	compound hetero	c.2380C>T:p.R794X	c.8268_8272del:p.2756_2758del	-	n.p.
RPKT0557	M	30	night blindness	62	SL+/SL+	sporadic	*EYS*	compound hetero	c.2528G>A:p.G843E	c.4957dupA:p.S1653fs	-	n.p.
RPKT0559	F	childhood	night blindness	55	0.2/0.4	AR	*EYS*	homo	c.7919G>A:p.W2640X	c.7919G>A:p.W2640X	-	n.p.
RPKT0560	F	68	night blindness	71	0.3/0.2	AR	*EYS*	compound hetero	c.6557G>A:p.G2186E	c.8805C>A:p.Y2935X	-	combined with NTG
RPKT0561	M	adolescence	constriction of VF	19	1.5/0.9	sporadic	*EYS*	compound hetero	c.4957dupA:p.S1653fs	c.8805C>A:p.Y2935X	-	n.p.
RPKT0570	M	20	night blindness	57	1.0/0.9	AR	*EYS*	compound hetero	c.4957dupA:p.S1653fs	c.5847C>A:p.Y1949X	-	n.p.
RPKT0575	M	childhood	night blindness	13	0.1/0.1	XL	*RP2*	hemi	c.28A>T:p.K10X	-	-	n.p.
RPKT0576	M	in his 3rd decades	night blindness	58	0.07/0.3	AR	*USH2A*	compound hetero	c.10859T>C:p.I3620T	c.12168G>A:p.W4056X	-	n.p.
RPKT0578	M	4	night blindness	5	0.3/0.3	sporadic	*RP1*	homo	c.4052_4053ins328	c.4052_4053ins328	-	n.p.
RPKT0579	F	childhood	night blindness	63	0.3/0.3	sporadic	*EYS*	compound hetero	c.4957dupA:p.S1653fs	c.7613delC:p.P2538fs	-	n.p.
RPKT0581	M	30	night blindness	44	1.2/1.2	sporadic	*EYS*	compound hetero	c.2528G>A:p.G843E	c.7919G>A:p.W2640X	-	n.p.
RPKT0585	M	20	night blindness	61	0.5/0.4	sporadic	*SAG*	homo	c.924delA:p.T308fs	c.924delA:p.T308fs	-	n.p.
RPKT0591	M	childhood	night blindness	67	SL-/0.06	XL	*RPGR*	hemi	c.2357_2358insGAGGAGAA:p.K786fs	-	-	combined with right eye RD
RPKT0593	M	50	decreased VA	76	0.3/0.15	AD	*SNRNP200*	hetero	c.3260C>T:p.S1087L	-	-	n.p.
RPKT0598	M	33	night blindness	81	HM/SL+	AR	*EYS*	homo	c.8805C>A:p.Y2935X	c.8805C>A:p.Y2935X	-	n.p.
RPKT0599	F	19	night blindness	54	0.2/0.2	AR	*EYS*	compound hetero	c.2528G>A:p.G843E	c.4957dupA:p.S1653fs	-	n.p.
RPKT0600	F	childhood	night blindness	36	0.9/1.0	sporadic	*EYS*	compound hetero	c.4957dupA:p.S1653fs	c.6557G>A:p.G2186E	-	n.p.
RPKT0602	M	67	night blindness	78	0.6/0.5	inconclusive	*RP1*	hetero	c.2143C>T:p.Q715X	-	-	n.p.
RPKT0603	M	childhood	night blindness	45	0.06/0.15	AR	*PRPF8*	hetero	c.6929G>A:p.R2310K	-	-	n.p.
RPKT0606	M	11	night blindness	58	0.03/0.1	AD	*PRPF31*	hetero	Gross deletion including exon 2 and 3***	-	-	n.p.
RPKT0613	F	adolescence	night blindness	27	1.2/1.2	sporadic	*EYS*	homo	c.2528G>A:p.G843E	c.2528G>A:p.G843E	-	n.p.
RPKT0620	M	adolescence	night blindness	44	0.05/0.01	inconclusive	*EYS*	compound hetero	c.4957dupA:p.S1653fs	c.8805C>A:p.Y2935X	-	n.p.
RPKT0626	M	30	night blindness	35	1.5/1.5	sporadic	*EYS*	compound hetero	c.2528G>A:p.G843E	c.4957dupA:p.S1653fs	-	n.p.
RPKT0628	F	30	constriction of VF	32	1.0/1.0	sporadic	*EYS*	compound hetero	c.4957dupA:p.S1653fs	c.6557G>A:p.G2186E	-	n.p.
RPKT0630	F	24	constriction of VF	26	1.5/1.5	sporadic	*EYS*	homo	c.2528G>A:p.G843E	c.2528G>A:p.G843E	-	n.p.
RPKT0633	M	childhood	night blindness	24	1.0/1.2	inconclusive	*REEP6*	compound hetero	c.230_233del:p.77_78del	c.280_281del:p.94_94del	-	n.p.
RPKT0636	M	childhood	night blindness	39	0.5/0.4	XL	*RPGR*	hemi	c.2706_2707del:p.902_903del	-	-	n.p.
RPKT0639	F	adolescence	decreased VA	70	SL-/SL+	sporadic	*CERKL*	homo	c.638dupT:p.L213fs	c.638dupT:p.L213fs	-	n.p.
RPKT0640	M	6	decreased VA	11	0.4/0.4	AR	*TULP1*	homo	c.1145T>C:p.F382S	c.1145T>C:p.F382S	-	n.p.
RPKT0641	M	27	night blindness	27	1.2/1.5	sporadic	*EYS*	homo	c.8805C>A:p.Y2935X	c.8805C>A:p.Y2935X	-	n.p.
RPKT0644	F	44	night blindness	46	1.5/1.5	sporadic	*EYS*	compound hetero	c.2528G>A:p.G843E	c.8805C>A:p.Y2935X	-	n.p.
RPKT0649	M	57	decreased VA	60	0.6/0.6	AR	*EYS*	compound hetero	c.4957dupA:p.S1653fs	c.6557G>A:p.G2186E	-	n.p.

NTG: normal tension glaucoma, PACS: primary angle closure syndrome, n.p.: nothing particular, p/o: pointed out, RD: retinal detachment, VF: visual field

*RPKT0467 was homozygous for two variants c.4196delG and c.6353G>A. **Gross deletion shown in Supplementary Figure1

***Complex rearrangement comprising gross deletion shown in Supplementary Figure2

Supplementary Table 3. Details of causative variants.

Chr.	Gene	Exon	Nucleotide change	Protein change	rsID	Variant type	MAF in databases			No. of damaging prediction	Satisfied criteria
							gnomAD	1000G	HGVD		
1	*RPE65*	14	c.1543C>T	p.R515W	rs121917745	missense	<0.0001	NA	NA	5/5	"DM" in HGMD
1	*USH2A*	3	c.490G>T	p.V164F	rs527236123	missense	NA	NA	NA	4/5	"DM" in HGMD
1	*USH2A*	13	c.2802T>G	p.C934W	rs201527662	missense	0.0002	0.0008	0.0008	5/5	"DM" in HGMD
1	*USH2A*	41	c.7660C>T	p.Q2554X	-	stopgain	NA	NA	NA	NA	likely pathogenic in ACMG guideline
1	*USH2A*	42	c.8254G>A	p.G2752R	rs201863550	missense	<0.0001	0.0004	NA	5/5	"DM" in HGMD
1	*USH2A*	50	c.9799T>C	p.C3267R	rs111033263	missense	<0.0001	NA	NA	5/5	"DM" in HGMD
1	*USH2A*	55	c.10859T>C	p.I3620T	rs779716464	missense	<0.0001	NA	0.0004	5/5	"DM" in HGMD
1	*USH2A*	62	c.12094G>A	p.G4032R	rs908265742	missense	<0.0001	NA	NA	5/5	"DM" in HGMD
1	*USH2A*	62	c.12168G>A	p.W4056X	-	stopgain	<0.0001	NA	NA	NA	likely pathogenic in ACMG guideline
1	*USH2A*	63	c.13010C>T	p.T4337M	rs527236137	missense	NA	NA	0.0004	4/5	"DM" in HGMD
1	*USH2A*	64	c.13847G>T	p.G4616V	rs527236124	missense	NA	NA	NA	5/5	"DM" in HGMD
2	*SNRNP200*	25	c.3260C>T	p.S1087L	rs267607077	missense	<0.0001	NA	NA	5/5	"DM" in HGMD
2	*CERKL*	4	c.638dupT	p.L213fs	-	fs_ins	NA	NA	NA	NA	pathogenic in ACMG guideline
2	*SAG*	11	c.924delA	p.T308fs	-	fs_del	NA	NA	0.0027	NA	likely pathogenic in ACMG guideline
3	*RHO*	1	c.302G>A	p.G101E	rs759945007	missense	<0.0001	NA	NA	5/5	"DM" in HGMD
4	*PDE6B*	1	c.51delT	p.D17fs	rs758552173	fs_del	NA	NA	NA	NA	likely pathogenic in ACMG guideline
4	*PDE6B*	12	c.1486delC	p.P496fs	-	fs_del	NA	NA	NA	NA	likely pathogenic in ACMG guideline
4	*PDE6B*	12	c.1604T>A	p.I535N	rs527236088	missense	<0.0001	NA	0.0009	4/5	"DM" in HGMD
4	*PDE6B*	13	c.1669C>T	p.H557Y	rs121918581	missense	<0.0001	NA	0.0004	5/5	"DM" in HGMD
4	*PDE6B*	14	c.1768delA	p.M590fs	-	fs_del	NA	NA	NA	NA	likely pathogenic in ACMG guideline
4	*PDE6B*	intron15	c.1920+2T>C	p.?	rs763996159	splicesite	<0.0001	NA	NA	NA	"DM" in HGMD
4	*CNGA1*	5	c.191delG	p.G64fs	rs527236058	fs_del	NA	NA	0.0012	NA	"DM" in HGMD
4	*CNGA1*	6	c.265delC	p.L89fs	rs749012133	fs_del	NA	NA	0.0012	NA	"DM" in HGMD
5	*PDE6A*	1	c.166G>T	p.E56X	rs772613637	stopgain	NA	NA	NA	NA	likely pathogenic in ACMG guideline
6	*EYS*	11	c.1750G>T	p.E584X	rs527236072	stopgain	NA	NA	NA	NA	"DM" in HGMD
6	*EYS*	15	c.2380C>T	p.R794X	rs371032798	stopgain	<0.0001	NA	NA	NA	"DM" in HGMD
6	*EYS*	16	c.2528G>A	p.G843E	rs74419361	missense	<0.0001	0.0014	0.022	4/5	"DM" in HGMD
6	*EYS*	23	c.3489T>A	p.N1163K	rs150951106	missense	0.0007	0.0018	0.0054	3/5	"DM?" in HGMD
6	*EYS*	25	c.3809T>G	p.V1270G	rs368856942	missense	<0.0001	0.0004	0.0045	0/5	"DM?" in HGMD
6	*EYS*	26	c.4897A>G	p.K1633E	rs77523865	missense	0.0001	0.0002	0.0021	1/5	"DM" in HGMD
6	*EYS*	26	c.4957dupA	p.S1653fs	rs527236065	fs_del	<0.0001	NA	0.0021	NA	"DM" in HGMD
6	*EYS*	28	c.5847C>A	p.Y1949X	-	stopgain	NA	NA	NA	NA	likely pathogenic in ACMG guideline
6	*EYS*	32	c.6557G>A	p.G2186E	rs527236068	missense	<0.0001	NA	NA	3/5	"DM" in HGMD
6	*EYS*	39	c.7609G>A	p.A2537T	rs189406424	missense	0.0002	0.0018	0.005	1/5	"DM?" in HGMD
6	*EYS*	39	c.7613delC	p.P2538fs	-	fs_del	NA	NA	NA	NA	likely pathogenic in ACMG guideline
6	*EYS*	39	c.7665_7666del	p.2555_2556del	rs368440268	fs_del	NA	NA	NA	NA	"DM" in HGMD
6	*EYS*	41	c.7919G>A	p.W2640X	rs527236066	stopgain	<0.0001	NA	NA	NA	"DM" in HGMD
6	*EYS*		6 kb deletion including the whole of exon 42		-	gross deletion	NA	NA	NA	NA	gross deletion/insertion
6	*EYS*	43	c.8268delC	p.S2756fs	rs775398034	fs_del	NA	NA	NA	NA	likely pathogenic in ACMG guideline
6	*EYS*	43	c.8268_8272del	p.2756_2758del	-	fs_del	<0.0001	NA	NA	NA	likely pathogenic in ACMG guideline
6	*EYS*	43	c.8650T>C	p.C2884R	-	missense	NA	NA	NA	4/5	"DM" in HGMD
6	*EYS*	43	c.8805C>A	p.Y2935X	rs527236067	stopgain	<0.0001	NA	0.0029	NA	"DM" in HGMD
6	*EYS*	43	c.8860T>C	p.F2954L	rs79036642	missense	0.0002	0.0008	0.0008	1/5	"DM" in HGMD
6	*EYS*	43	c.8868delT	p.T2956fs	-	fs_del	NA	NA	NA	NA	likely pathogenic in ACMG guideline
6	*MAK*	15	c.1825C>T	p.Q609X	-	stopgain	NA	NA	NA	NA	likely pathogenic in ACMG guideline
6	*TULP1*	12	c.1145T>C	p.F382S	rs121909076	missense	<0.0001	NA	0.0008	5/5	"DM" in HGMD
8	*RP1L1*	2	c.324_325insT	p.P109fs	rs753986463	fs_ins	0.0019	NA	0.0167	NA	"DM" in HGMD
8	*RP1L1*	4	c.3026_3029del	p.1009_1010del	-	fs_del	NA	NA	NA	NA	likely pathogenic in ACMG guideline
8	*RP1*	4	c.2143C>T	p.Q715X	-	stopgain	NA	NA	NA	NA	likely pathogenic in ACMG guideline
8	*RP1*	4	c.4052_4053ins328	p.Y1352A fsX9	-	fs_ins	NA	NA	NA	NA	*Alu* insertion in *RP1*
8	*RP1*	4	c.4196delG	p.C1399fs	rs201839635	fs_del	<0.0001	NA	NA	NA	"DM" in HGMD
8	*RP1*	4	c.5797C>T	p.R1933X	rs118031911	stopgain	0.0002	0.0004	0.0033	NA	likely pathogenic in ACMG guideline
8	*RP1*	4	c.6353G>A	p.S2118N	rs753732597	missense	<0.0001	NA	NA	1/5	"DM" in HGMD
17	*PRPF8*	36	c.5792C>T	p.T1931M	-	missense	NA	NA	NA	5/5	"DM?" in HGMD
17	*PRPF8*	43	c.6926A>C	p.H2309P	rs121434236	missense	NA	NA	NA	5/5	"DM" in HGMD
17	*PRPF8*	43	c.6929G>A	p.R2310K	rs121434238	missense	NA	NA	NA	5/5	"DM" in HGMD
19	*REEP6*	3	c.230_233del	p.77_78del	rs781429331	fs_del	NA	NA	NA	NA	likely pathogenic in ACMG guideline
19	*REEP6*	3	c.280_281del	p.94_94del	rs759026615	fs_del	<0.0001	NA	NA	NA	pathogenic in ACMG guideline
19	*PRPF31*		70 kb deletion including the whole exon1&2		-	gross deletion	NA	NA	NA	NA	gross deletion/insertion
19	*PRPF31*	4	c.316G>T	p.E106X	-	stopgain	NA	NA	NA	NA	likely pathogenic in ACMG guideline
X	*RPGR*	7	c.764C>T	p.T255I	-	missense	NA	NA	NA	5/5	"DM" in HGMD
X	*RPGR*	8	c.793dupA	p.T265fs	-	fs_ins	NA	NA	NA	NA	likely pathogenic in ACMG guideline
X	*RPGR*	14	c.1665delA	p.E555fs	-	fs_del	NA	NA	NA	NA	likely pathogenic in ACMG guideline
X	*RPGR*	15	c.2236_2237del	p.746_746del	-	fs_del	NA	NA	NA	NA	likely pathogenic in ACMG guideline
X	*RPGR*	15	c.2357_2358insGAGGAGAA	p.K786fs	-	fs_ins	NA	NA	NA	NA	likely pathogenic in ACMG guideline
X	*RPGR*	15	c.2706_2707del	p.902_903del	-	fs_del	NA	NA	NA	NA	likely pathogenic in ACMG guideline
X	*RP2*	1	c.28A>T	p.K10X	-	stopgain	NA	NA	NA	NA	likely pathogenic in ACMG guideline

fs_del : frameshift deletion, fs_ins : frameshift insertion, NA : not applicable

Supplementary Table 4.
Details of possibly solved patients, causative variants and VUS.
NOTICE : The variants need to be replicated in further ethnic-matched study for confirmation of pathogenicity before the patients are recruited to any available clinical trials of gene therapies.

SampleID	Sex	Age of onset	First subjective symptom	Age of first visit to our institute	VA at first visit to our institute	Hereditary trait indicated from pedigree tree	Gene	Genotype	Variant 1	Variant 2	Comment on phenotype
RPKT0343	F	40	p/o at occasional medical exam	40	1.5/1.5	inconclusive	*CNGA1*	homo	c.41A>T:p.Q14L	c.41A>T:p.Q14L	atypical phenotype(dystrophy only in nasal area)
RPKT0354	F	55	decreased VA	82	1.2/1.2	inconclusive	*IFT140*	compound hetero	c.2542C>T:p.R848C	c.3018_3019del:p.1006_1007del	n.p.
RPKT0368	F	13	night blindness	13	1.5/1.5	sporadic	*CACNA1F*	compound hetero	c.3334G>T:p.E1112X	c.3346T>G:p.F1116V	n.p.
RPKT0371	F	in her 7th decades	night blindness	75	0.4/0.5	inconclusive	*EYS*	compound hetero	c.2528G>A:p.G843E	c.525_527del:p.175_176del	n.p.
RPKT0391	M	20	night blindness	46	0.7/1.0	inconclusive	*RHO*	hetero	c.539C>T:p.P180L	-	conbined with CSC
RPKT0494	M	61	constriction of VF	61	1.0/1.2	AR	*GPR98*	compound hetero	c.940G>A:p.A314T	c.3046G>A:p.E1016K	atypical phenotype(without pigemntation)
RPKT0503	M	60	decreased VA	63	1.2/1.2	sporadic	*GUCY2D*	hetero	c.162C>G:p.F54L	-	n.p.
RPKT0508	M	10	p/o at occasional medical exam	18	SL-/0.5	sporadic	*SNRNP200*	hetero	c.2594G>A:p.G865D	-	combined with right eye ECD and RD
RPKT0520	M	childhood	night blindness	41	0.9/0.7	AR	*PDE6B*	hetero	c.1514A>C:p.H505P	-	n.p.
RPKT0547	F	59	night blindness	68	0.2/0.2	sporadic	*ZNF408*	homo	c.377G>A:p.S126N	c.377G>A:p.S126N	n.p.
RPKT0550	F	in her 4th decades	night blindness	59	0.6/0.5	sporadic	*TOPORS*	hetero	c.2078G>T:p.R693I	-	n.p.
RPKT0555	M	adolescence	night blindness	26	0.7/0.7	AR	*CLN3*	homo	c.1007C>A:p.S336Y	c.1007C>A:p.S336Y	atypical phenotype(without pigmentation)
RPKT0568	F	in his 3rd decades	night blindness	56	0.2/0.3	sporadic	*MAK*	compound hetero	c.512T>C:p.L171S	c.1825C>T:p.Q609X	n.p.
RPKT0569	M	52	p/o at occasional medical exam	59	1.0/1.0	AR	*USH2A*	homo	c.10967T>C:p.F3656S	c.10967T>C:p.F3656S	n.p.
RPKT0610	F	65	decreased VA	72	0.01/0.3	sporadic	*CRB1*	hetero	c.3131C>T:p.P1044L	-	atypical phenotype(mainly paravenous pigement)
RPKT0611	M	51	p/o at occasional medical exam	54	1.5/1.5	sporadic	*USH2A*	compound hetero	c.12863C>A:p.P4288Q	c.12863C>A:p.P4288Q	n.p.
RPKT0614	M	45	night blindness	56	1.2/1.2	sporadic	*USH2A*	compound hetero	c.13847G>T:p.G4616V	c.15355C>T:p.R5119W	n.p.
RPKT0615	F	childhood	night blindness	43	0.4/0.4	sporadic	*USH2A*	compound hetero	c.14286C>G:p.N4762K	c.14243C>T:p.S4748F	n.p.
RPKT0621	M	52	constriction of VF	58	0.4/0.8	AR	*PRPF8*	hetero	c.6345C>G:p.I2115M	-	n.p.
RPKT0632	M	**not recorded**	**not recorded**	70	0.1/0.03	inconclusive	*SNRNP200*	hetero	c.1532A>C:p.N511T	-	n.p.
RPKT0647	M	20	night blindness	21	1.2/1.5	sporadic	*EYS*	compound hetero	c.4957dupA:p.S1653fs	c.6563T>C:p.I2188T	n.p.
RPKT0650	F	childhood	night blindness	54	0.3/0.3	sporadic	*FLVCR1*	compound hetero	c.368T>G:p.F123C	c.733A>G:p.N245D	n.p.

p/o: pointed out, VA : visual acuity, VF: visual field, n.p.: nothing particular, CSC: central serous chorioretinopathy,
ECD: endothelial corneal dystrophy, RD: retinal detachment

Supplementary Table 5.

Details of candidate causative variants evaluated as VUS in the current study.

NOTICE : These variants need to be replicated in further ethnic-matched study for confirmation of pathogenicity.

Chr.	Gene	Exon	Nucleotide change	Protein change	rsID	Variant type	MAF in databases			No. of damaging prediction
							gnomAD	1000G	HGVD	
1	*CRB1*	9	c.3131C>T	p.P1044L	-	missense	NA	NA	NA	3/5
1	*FLVCR1*	1	c.368T>G	p.F123C	-	missense	NA	NA	NA	4/5
1	*FLVCR1*	1	c.733A>G	p.N245D	-	missense	NA	NA	NA	5/5
1	*USH2A*	56	c.10967T>C	p.F3656S	-	missense	NA	NA	NA	4/5
1	*USH2A*	63	c.12863C>A	p.P4288Q	-	missense	NA	NA	NA	5/5
1	*USH2A*	65	c.14286C>G	p.N4762K	rs750368946	missense	<0.0001	NA	NA	4/5
1	*USH2A*	71	c.15355C>T	p.R5119W	rs767137840	missense	0.0001	NA	0.0004	2/5
2	*SNRNP200*	20	c.2594G>A	p.G865D	-	missense	NA	0.0004	NA	5/5
2	*SNRNP200*	13	c.1532A>C	p.N511T	-	missense	NA	NA	NA	5/5
3	*RHO*	3	c.539C>T	p.P180L	-	missense	NA	0.0008	NA	5/5
4	*PDE6B*	12	c.1514A>C	p.H505P	-	missense	NA	NA	NA	4/5
4	*CNGA1*	4	c.41A>T	p.Q14L	-	missense	NA	NA	0.0004	0/5
5	*GPR98*	7	c.940G>A	p.A314T	rs533540279	missense	<0.0001	NA	0.0021	3/5
5	*GPR98*	17	c.3046G>A	p.E1016K	-	missense	NA	NA	NA	5/5
6	*MAK*	7	c.512T>C	p.L171S	-	missense	NA	NA	0.0012	5/5
6	*EYS*	4	c.525_527del	p.E175del	rs780433094	nonfs_del	9.55E-05	NA	0.0008	NA
6	*EYS*	32	c.6563T>C	p.I2188T	rs1562190751	missense	<0.0001	NA	NA	2/5
9	*TOPORS*	3	c.2078G>T	p.R693I	-	missense	NA	NA	NA	3/5
11	*ZNF408*	3	c.377G>A	p.S126N	rs536561101	missense	<0.0001	0.0002	0.0041	3/5
16	*IFT140*	20	c.2542C>T	p.R848C	rs201384469	missense	0.0003	NA	0.0006	4/5
16	*CLN3*	14	c.1007C>A	p.S336Y	rs761795794	missense	NA	NA	NA	4/5
17	*PRPF8*	39	c.6345C>G	p.I2115M	-	missense	NA	NA	NA	4/5
17	*GUCY2D*	2	c.162C>G	p.F54L	-	missense	NA	NA	NA	4/5
X	*CACNA1F*	28	c.3334G>T	p.E1112X	-	stopgain	NA	NA	NA	NA
X	*CACNA1F*	28	c.3346T>G	p.F1116V	-	missense	NA	NA	NA	5/5

nonfs_del : nonframeshift deletion, NA : not applicable

Supplementary Figure 1. Heterozygous deletion spanning exon 42 of *EYS*.

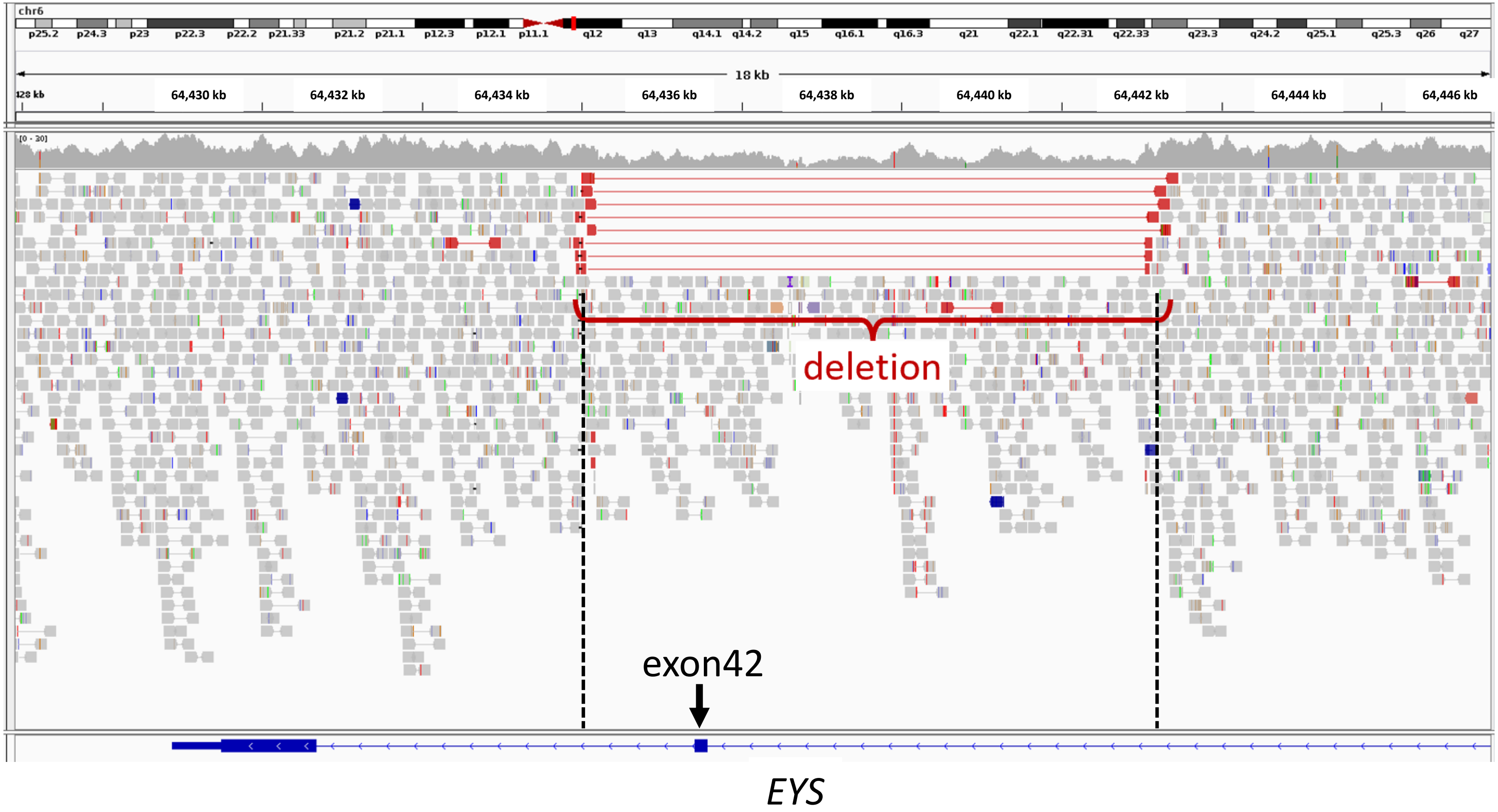

Read-depth analysis shows heterozygous large deletion (red) that includes all of exon 42
in patient with founder mutation c.4957dupA

Supplementary Figure 2. Complex rearrangements including *PRPF31*.

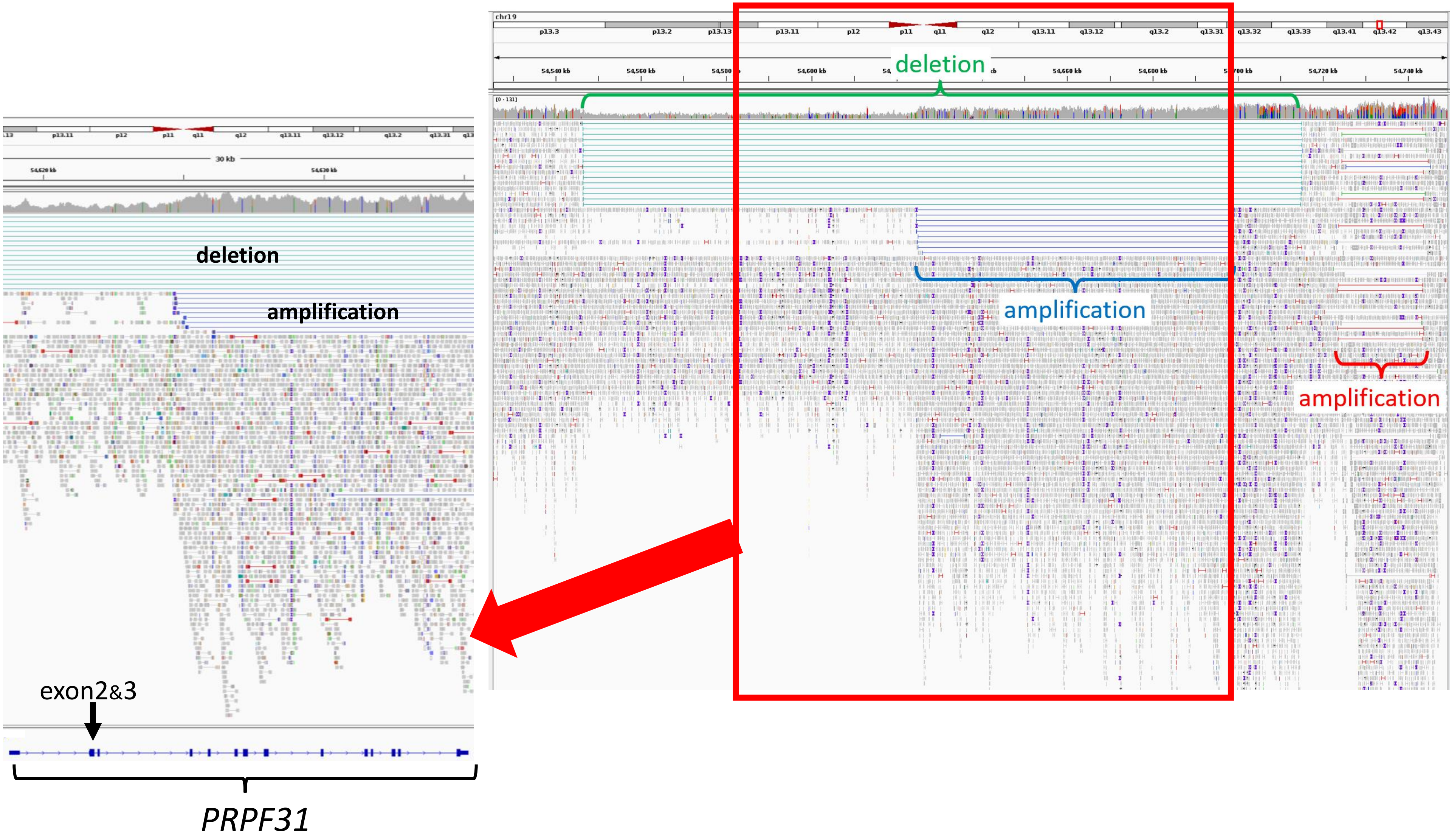

Read-depth analysis shows complex rearrangement on long arm of chromosome 19 comprising 160 kb deletion (green) including protein-coding exons 2 and 3 of *PRPF31* and two amplified regions (blue and red)

Supplementary Figure 3.

Atypical phenotype of patient possibly solved with VUS of *CNGA1*.

NOTICE : The patient’s variants need to be replicated in further ethnic-matched study for confirmation of pathogenicity before any available clinical trials of gene therapies.

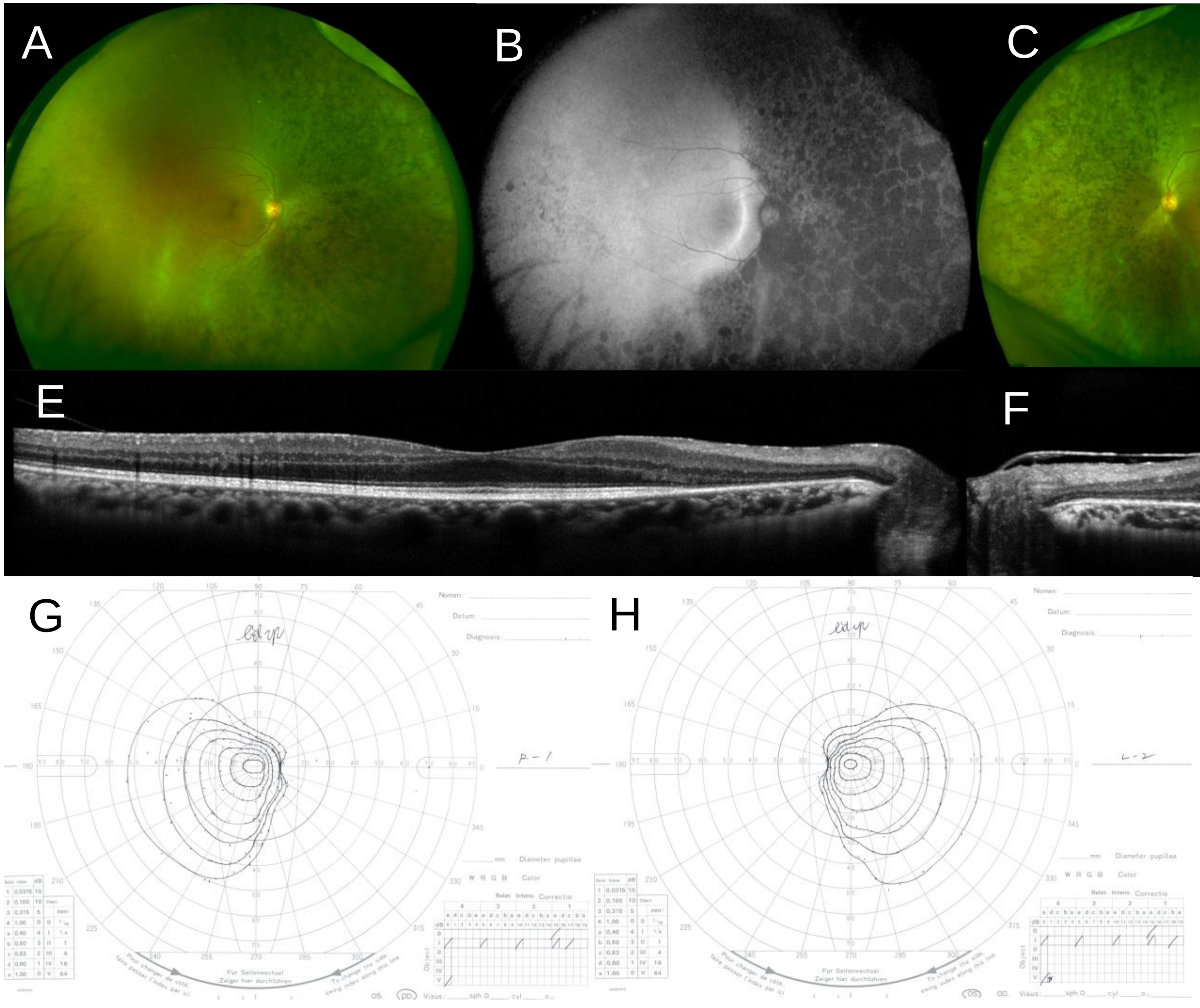

A,C: Color fundus photograph by Optos® 200Tx
B,D: Fundus autofluorescence
E,F: Horizontal crossectional images by Spectralis®
G,H: Viual field testing by Goldmann-Perimeter

Supplementary Figure 4.

Atypical phenotype of patient possibly solved with VUS of *GPR98*.

NOTICE : The patient's variants need to be replicated in further ethnic-matched study for confirmation of pathogenicity before any available clinical trials of gene therapies.

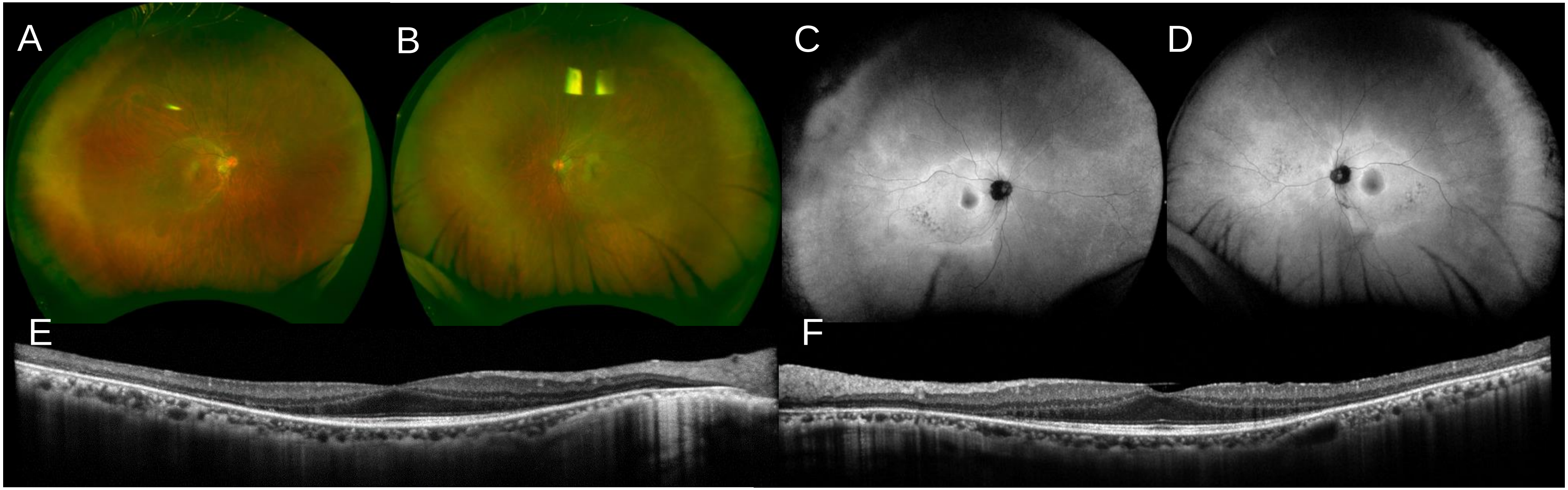

A,C: Color fundus photograph by Optos® 200Tx B,D: Fundus autofluorescence
E,F: Horizontal crossectional images by Spectralis®

Supplementary Figure 5.

Atypical phenotype of patient possibly solved with VUS of *CRB1*.

NOTICE : The patient's variants need to be replicated in further ethnic-matched study for confirmation of pathogenicity before any available clinical trials of gene therapies.

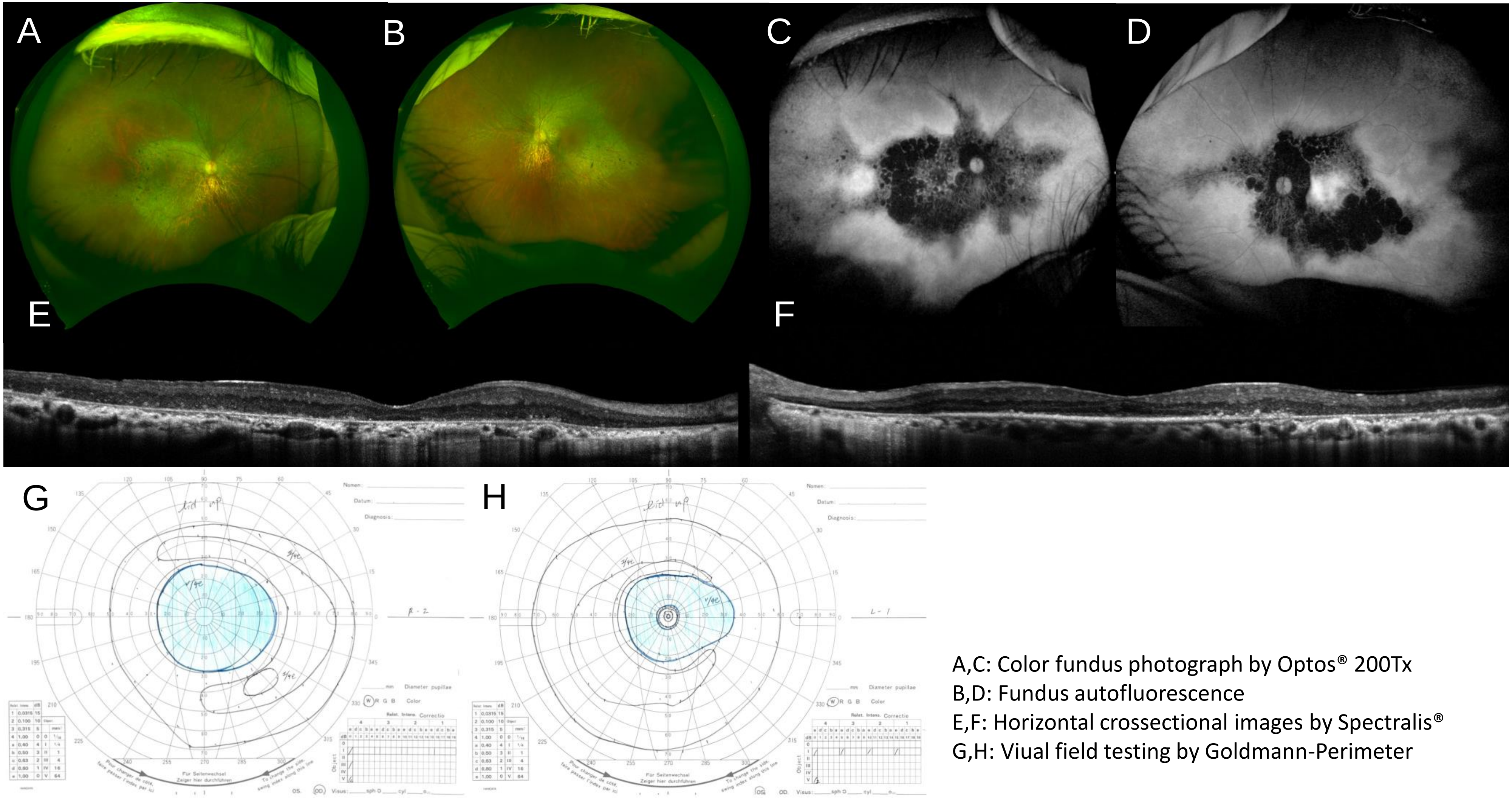

A,C: Color fundus photograph by Optos® 200Tx
B,D: Fundus autofluorescence
E,F: Horizontal crossectional images by Spectralis®
G,H: Viual field testing by Goldmann-Perimeter

Supplementary Figure 6.

Atypical phenotype of patient possibly solved with VUS of *CLN3*.

NOTICE : The patient's variants need to be replicated in further ethnic-matched study for confirmation of pathogenicity before any available clinical trials of gene therapies.

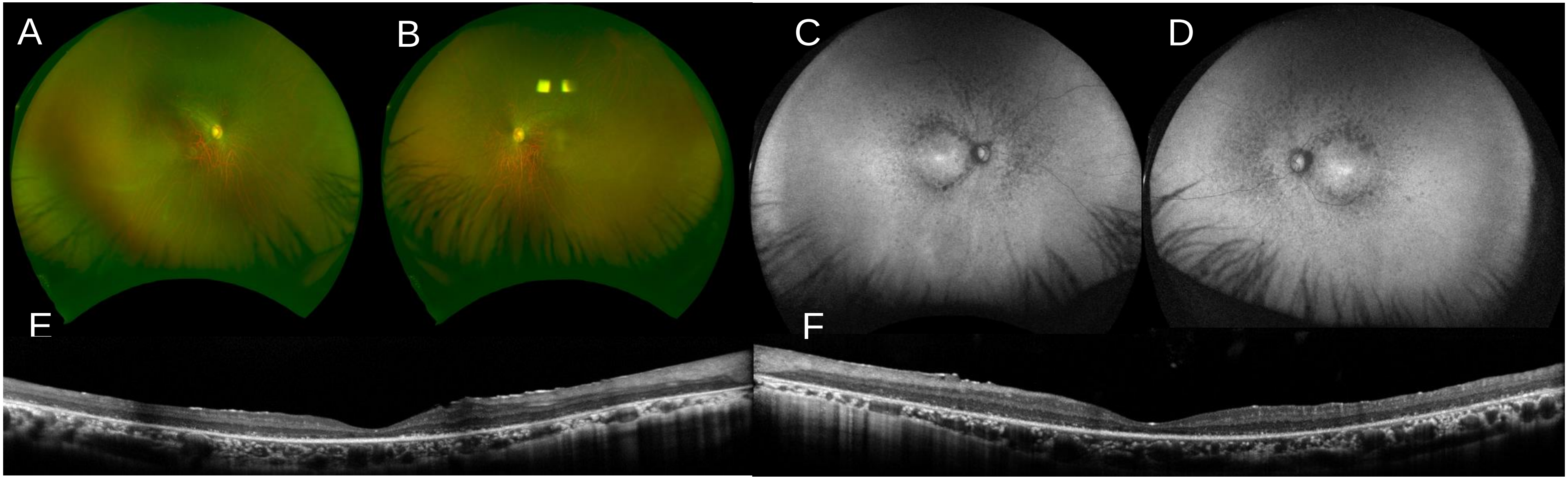

A,C: Color fundus photograph by Optos® 200Tx B,D: Fundus autofluorescence
E,F: Horizontal crossectional images by Spectralis®

Supplementary Figure 7.

Phenotype of the patient possibly solved with VUS of *GUCY2D*.

NOTICE : The patient's variants need to be replicated in further ethnic-matched study for confirmation of pathogenicity before any available clinical trials of gene therapies.

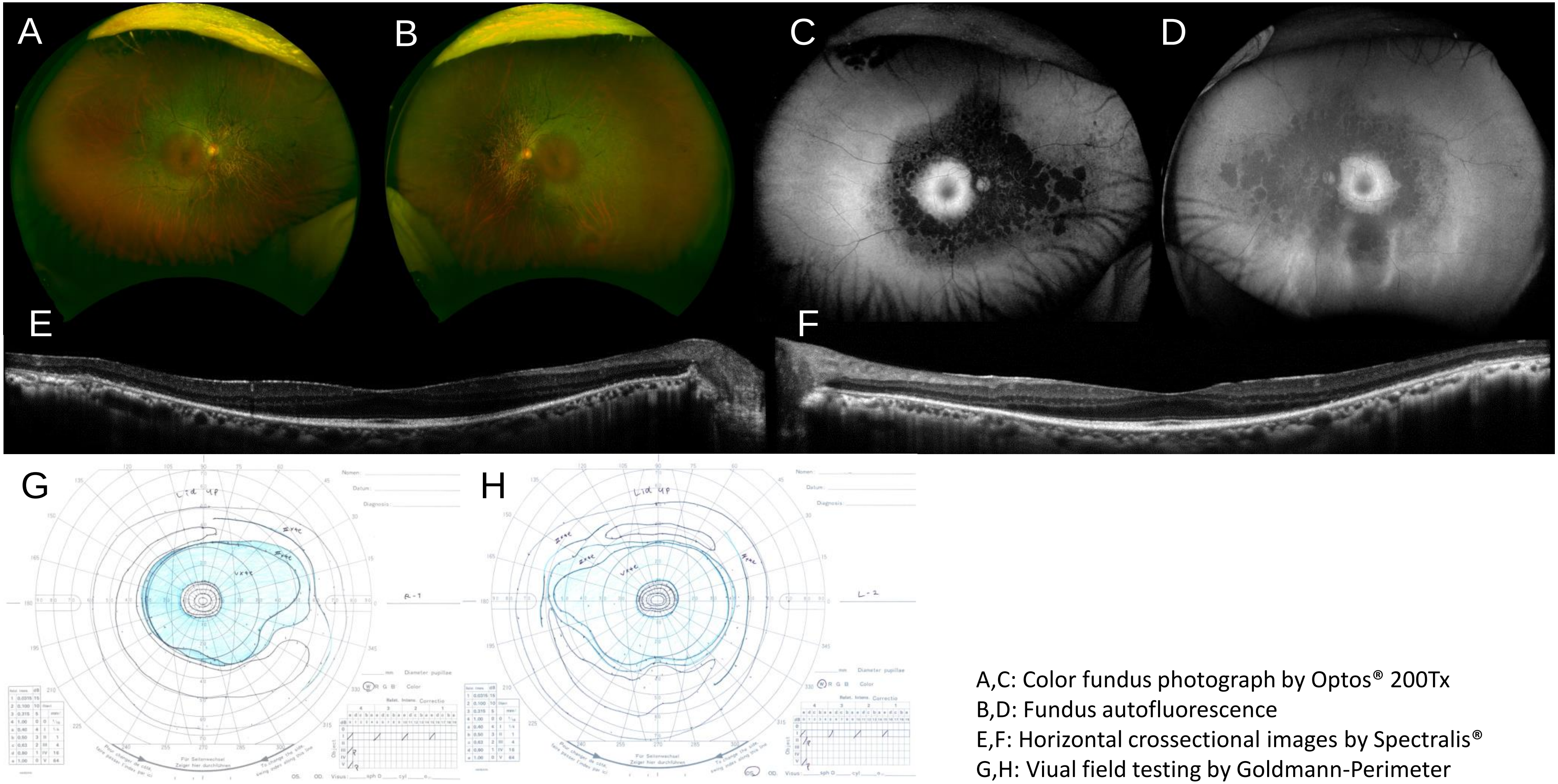

A,C: Color fundus photograph by Optos® 200Tx
B,D: Fundus autofluorescence
E,F: Horizontal crossectional images by Spectralis®
G,H: Viual field testing by Goldmann-Perimeter
